# Identification of New Biomarker for Prediction of Hepatocellular Carcinoma Development in Early-Stage Cirrhosis Patients

**DOI:** 10.1155/2021/9949492

**Published:** 2021-07-20

**Authors:** Gang Ning, Yongqiang Li, Wenji Chen, Wenjuan Tang, Diwen Shou, Qingling Luo, Huiting Chen, Yongjian Zhou

**Affiliations:** Department of Gastroenterology and Hepatology, Guangzhou Digestive Diseases Center, Guangzhou First People's Hospital, South China University of Technology, Guangzhou, Guangdong Province, China

## Abstract

**Background:**

Liver cirrhosis is one of the major drivers of hepatocellular carcinoma (HCC). In the present study, we aimed to identify and validate new biomarker for early prediction of HCC development in early-stage cirrhosis patients.

**Methods:**

mRNA expression and clinical parameters of GSE63898, GSE89377, GSE15654, GSE14520, and TCGA-HCC cohort and ICGC-HCC cohort were downloaded for analysis. Wilcoxon test was performed to identify DEGs. Univariate and multivariate Cox regression analysis were used to develop the risk signature, and ROC analysis was performed to analyze the predictive accuracy and sensitivity of the risk signature.

**Results:**

There were 42 DEGs (including 28 upregulated genes and 14 downregulated genes) found in early-stage liver cirrhosis patients before developing HCC from GSE1565442. Then, a risk signature consisting of 8 DEGs could effectively classify early-stage cirrhosis patients into high-risk group with shorter HCC development time and low-risk group with longer HCC development time from GSE15654. Multivariate Cox analysis indicated that the risk signature was an independent prognostic factor for the prediction of HCC development and ROC analysis showed that the signature exhibited good predictive efficiency in predicting 2-, 5-, and 10-year HCC development. Mechanistically, significantly higher proportions of CD8 T cells were found to be enriched in cirrhosis patients with low risk score, and higher CD8 T cells were associated with longer HCC development time. Besides, the signature was an independent prognostic factor for poorer prognosis of early-stage liver cirrhosis patients of GSE15654. Moreover, the signature could also separate HCC patients from healthy controls and was also associated with the poorer prognosis of HCC patients from three HCC cohorts. Finally, we also identified HDAC inhibitors, such as trichostatin A, to be a potential chemopreventive treatment for the prevention of HCC development by targeting risk signature based on CMap analysis.

**Conclusion:**

A risk signature was developed and validated for early prediction of HCC development, which may be a useful tool to set up individualized follow-up interval schedules.

## 1. Introduction

Hepatocellular carcinoma (HCC) continues to be a serious threat and burden to public health as it represents the sixth most common cancers and the fourth leading cause of cancer-related death worldwide [1]. Moreover, the incidence of HCC has increased rapidly in regions, previously identified as lower rates, such as Oceania, Western Europe, and Northern America [2]. Much achievement has been made in treating HCC. However, the disease is still tackled with low remission rate, high recurrence, and low survival rate. To date, no effective adjuvant therapy is available for prevention of recurrence [3, 4]. Furthermore, at times of the diagnosis of HCC is made, 40% to 50% of the patients are at their advanced stages and the treatment options are very limited [5]. This comes to great clinical urgency in identification of a potential new efficient method for the diagnosis of HCC.

HCC is known to be related to inflammation and liver damage [6]. Liver cirrhosis is a notable risk factor for HCC and is often found prior to the development of HCC [7, 8]. The incidence of HCC in non-cirrhosis patients is 0.5% to 1.0%, but the incidence of HCC in cirrhosis patients reaches as much as 3%–6%. Current practice guideline suggests the necessity of regular HCC surveillance [9–12]. However, there were up to 12% of HCC patients could be diagnosed through current surveillance recommendation [13]. Other effective prognostic signature is needed to identify cirrhosis patients who are at high risk for HCC development and should be further justified.

Recent applications of microarray and high-throughput technologies have provided a better method to better understand the molecular mechanism of the transformation from liver cirrhosis to HCC [14, 15], through which new biomarkers in screening HCC in cirrhosis patients could be explored. In the present study, we first screened out differentially expressed genes (DEGs) between cirrhosis patients and early HCC patients and then further identified those DEGs whose expression had been altered in early-stage liver cirrhosis patients before development of HCC. Next, we developed and validated a risk signature for prediction of HCC development for early-stage liver cirrhosis patients and analyzed the association of the risk signature with infiltrating immune cells. Besides, we assessed the prognostic value of this signature in HCC patients. Moreover, we sought to identify potential bioactive compounds which could be potential chemopreventive treatment for the prevention of HCC development by targeting the risk signature.

## 2. Materials and Methods

### 2.1. Ethics Statement

All the data used for analysis in the present study were downloaded from The Cancer Genome Atlas (TCGA), Gene Expression Omnibus (GEO), and International Cancer Genome Consortium (ICGC) database, so written consents had already been obtained before our study.

### 2.2. Data Acquisition

mRNA expression profiles of GSE638 98, GSE89377, GSE15654, and GSE14520 were downloaded from GEO database (https://www.ncbi.nlm.nih.gov/geo/). mRNA expression profile of TCGA-HCC cohort was got from GDC Data portal (https://cancergenome.nih.gov/). mRNA expression profile of ICGC Japan HCC cohort was attained from the ICGC portal (https://dcc.icgc.org/projects/LIRI-JP). All the data were log2-trasformed for data normalization.

In GSE63898, there were 196 early HCC patients (defined as BCLC 0/A stage) and 168 liver cirrhosis patients. In GSE89377, there were 12 liver cirrhosis patients, 22 patients with dysplastic nodules, 5 early HCC patients, and 23 advanced HCC patients. GSE63898 and GSE89377 were used to identify DEGs and KEGG pathways between liver cirrhosis patients and early HCC patients. DEGs were identified with a cut-off value of *p* < 0.05. KEGG pathways enriched by these DEGs were identified in DAVID (https://david.ncifcrf.gov/summary.jsp), and a cut-off value of *p* < 0.05 was considered as statistically significant.

In GSE15654, gene expression profile of formalin-fixed needle biopsy specimens from the livers of 216 patients with hepatitis C-related early-stage (Child–Pugh class A) cirrhosis were available. These patients were prospectively followed up for a median of 10 years at an Italian center, and there were 65 liver cirrhosis who finally developed HCC. GSE15654 was used to identify the DEGs and KEGG pathways between liver cirrhosis patients who would or would not develop HCC with similar method as described above. Besides, GSE15654 was also used to develop a risk signature for prediction of HCC development for early-stage liver cirrhosis patients. Basic characteristics of 216 patients with early-stage cirrhosis were summarized in Table 1.

Three HCC cohorts, including TCGA-HCC cohort (377 HCC patients and 50 normal controls), GSE14520 HCC cohort (220 HCC patients), and ICGC HCC cohort (232 HCC patients), were used to examine the prognostic value of the risk signature in HCC patients. Basic characteristics of HCC patients from these three cohorts were summarized in Table 2.

### 2.3. Development and Validation of Risk Signature for Early Prediction of HCC Development for Liver Cirrhosis Patients

First, these 216 early-stage liver cirrhosis patients were randomly divided into training cohort (*N* = 108) and validation cohort (*N* = 108) with an allocation of 1 : 1. Next, univariate and multivariate Cox regression analysis were performed to screen out the HCC development-associated genes in the training cohort. Then, a risk signature was constructed based on the coefficients weighted by multivariate Cox regression analysis. With the help of this signature, risk score for each patient was calculated and they were divided into high-risk group and low-risk group with the best cut-off value calculated by X-Tile software (http://tissuearray.org/, version 3.6.1). After that, the prognostic and predictive values of this signature were analyzed by univariate and multivariate Cox regression analysis and time-dependent ROC analysis. Finally, the applicability of the signature was also validated in the validation cohort and the whole cohort.

### 2.4. CIBORSORT

CIBERSORT (https://cibersort.stanford.edu), an online tool designed for estimating the abundances of infiltrating immune cells, was used for estimating infiltrating immune cells of 216 liver cirrhosis patients on the basis of mRNA expression profiles [16].

### 2.5. CMap Analysis

HCC development-associated genes identified by multivariate Cox regression analysis were classified into upregulated genes with the HR > 1 and downregulated genes with the HR < 1. Next, these genes were uploaded to the CMap web tool (https://portals.broadinstitute.org) to screen out compounds that may be potential chemopreventive treatment for the prevention of HCC development. Scores that ranged from −1 to 1 represented the correlation between compounds and risk signature genes. A negative score indicated that the corresponding compounds could reverse the expression of related genes and thus may be more likely to be used for chemopreventive treatment for prevention of HCC development, and vice versa [17].

### 2.6. Data Analysis Flowchart

**Figure 1:**
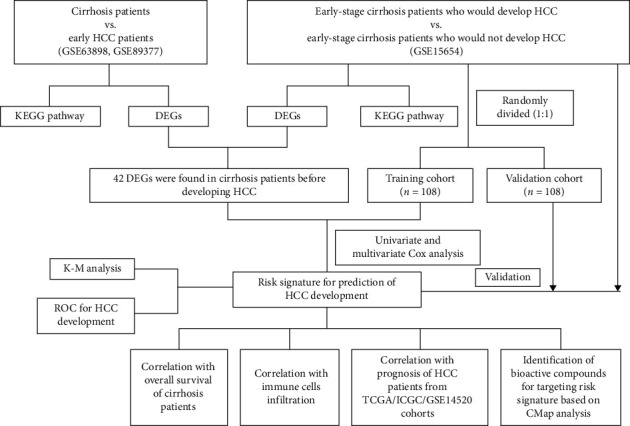
The workflow chart of the present study.

A workflow of the study was depicted and is shown in Figure 1.

### 2.7. Statistical Analysis

R software (version 3.5.1) and GraphPad Prism 6 (GraphPad Software, La Jolla, CA, USA) were used for statistical analysis. Wilcoxon test was performed to identify DEGs between patients with liver cirrhosis and early HCC patients, or early-stage liver cirrhosis patients who would or would not develop HCC. Unpaired Student's *t*-tests or ANOVA tests were performed to compare the difference of risk score between two groups or more than three groups. Kaplan–Meier analysis with two-side log-rank test was performed to analyze the difference of HCC development time, overall survival (OS), or disease-free survival (DFS) between patients of different risk scores. Univariate and multivariate Cox regression analysis were performed to analyze the prognostic value of the risk signature. Time-dependent ROC was performed to analyze the predictive accuracy and sensitivity of the risk signature. Additional statistical analyses were performed with STAMP [18]. *p* < 0.05 was considered as statistically significant.

## 3. Results

### 3.1. DEGs and KEGG Pathways between Liver Cirrhosis Patients and Early HCC Patients

**Figure 2:**
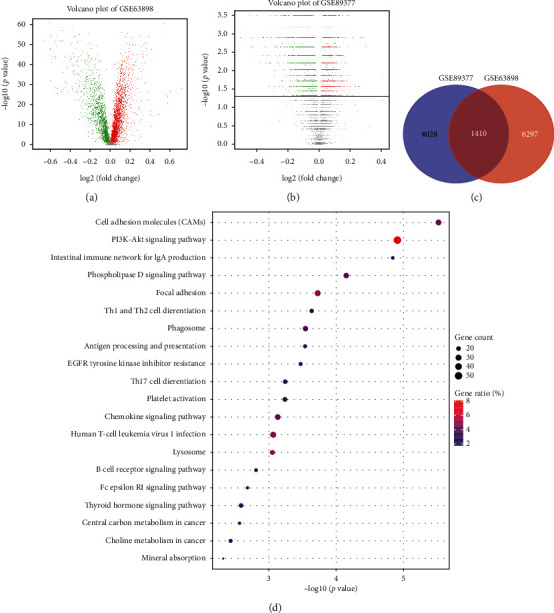
Differentially expressed genes (DEGs) and KEGG pathways between liver cirrhosis patients and early HCC patients. (a) Volcano figure of DEGs identified between liver cirrhosis patients and early HCC patients of GSE63898. (b) Volcano figure of DEGs identified between liver cirrhosis patients and early HCC patients of GSE89377. (c) Venn diagram of overlapped DEGs from GSE63898 and GSE89377. (d) KEGG pathways enriched in early HCC patients.

First, 7707 DEGs, including 4288 upregulated genes and 3419 downregulated genes, were found at early HCC patients (BCLC 0/A stage) compared to liver cirrhosis patients from GSE63898 (Figure 2(a)). Next, in order to reduce the selection bias, DEGs between 12 liver cirrhosis patients and 5 early HCC patients from GSE89377 were also identified, and 5438 DEGs, including 2782 upregulated genes and 2656 downregulated genes, were identified in early HCC patients compared to liver cirrhosis patients (Figure 2(b)). In total, 1410 DEGs, including 662 upregulated genes and 748 downregulated genes, were identified in early HCC patients by overlapping DEGs from GSE63898 and GSE89377 (Figure 2(c), Supplementary Material 1). These 1410 DEGs were mainly enriched in KEGG pathways, such as cell adhesion molecules (CAMs), PI3K-Akt signaling pathway, focal adhesion, antigen processing and presentation, chemokine signaling pathway, central carbon metabolism in cancer, and choline metabolism in cancer (Figure 2(d)), which had been found to be related with the development and progression of HCC. These results indicated that the above DEGs and KEGG pathways may play an important role in the transformation of liver cirrhosis to early HCC.

### 3.2. DEGs and KEGG Pathways between Early-Stage Liver Cirrhosis Patients Who Would or Would Not Develop HCC

**Figure 3:**
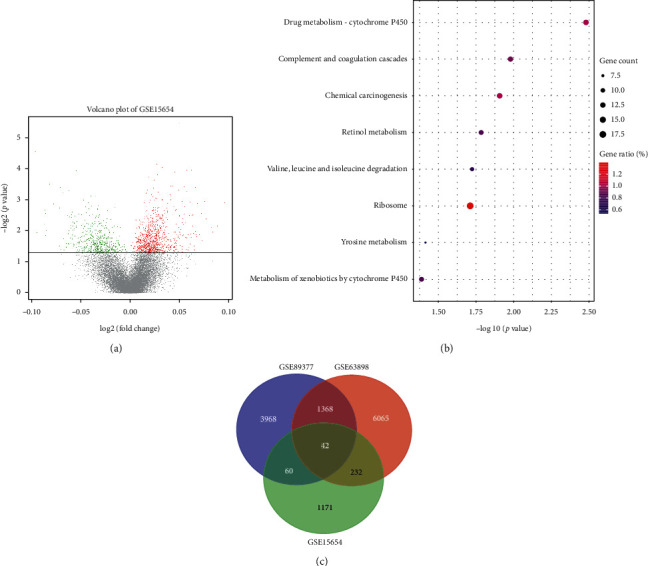
DEGs and KEGG pathways between early-stage liver cirrhosis patients who would or would not develop HCC of GSE15654. (a) Volcano figure of DEGs between early-stage liver cirrhosis patients who would or would not develop HCC of GSE15654. (b) KEGG pathways enriched in early-stage liver cirrhosis patients who would develop HCC. (c) Venn diagram of overlapped DEGs from GSE63898 and GSE89377 and GSE15654.

After identifying the DEGs and KEGG pathways between liver cirrhosis patients and early HCC patients, we next aimed to identify the DEGs and KEGG pathways between early-stage liver cirrhosis patients who would or would not develop HCC. In total, 1511 DEGs, including 972 upregulated genes and 539 downregulated genes, were found at liver cirrhosis patients who developed HCC compared to liver cirrhosis patients who would not develop HCC (Figure 3(a)). These DEGs were mainly enriched in KEGG pathways, such as complement and coagulation cascades, chemical carcinogenesis, retinol metabolism, valine, leucine and isoleucine degradation, tyrosine metabolism, and metabolism of xenobiotics by cytochrome P450 (Figure 3(b)). Compared to the KEGG pathways enriched in early HCC patients, abnormal metabolism of nutrient substance, such as carbon, choline, and amino acid, had been found at early-stage liver cirrhosis patients who would develop HCC. Moreover, compared to the DEGs identified in early HCC patients, 42 DEGs, including 28 upregulated genes and 14 downregulated genes, were found to be already abnormal in early-stage liver cirrhosis patients before developing HCC (Figure 3(c), Supplementary Material 2), suggesting that these abnormally expressed genes may be served as biomarkers for the identification of early-stage liver cirrhosis patients who were at high risk for HCC development.

### 3.3. Construction of a Risk Signature for Early Prediction of HCC Development for Early-Stage Liver Cirrhosis Patients of the Training Cohort

**Figure 4:**
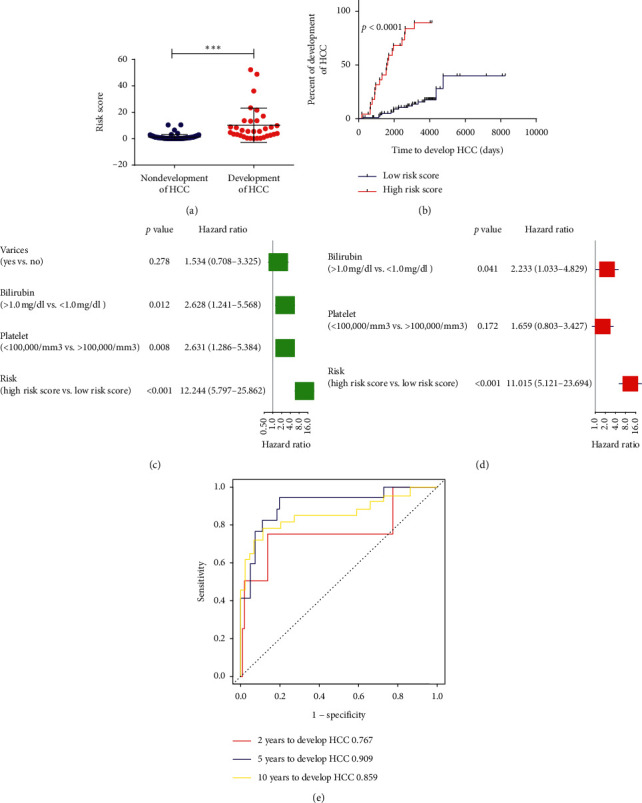
Construction of a risk signature for prediction of HCC development for early-stage liver cirrhosis patients of the training cohort. (a) Risk score between early-stage liver cirrhosis patients who would or would not develop HCC. (b) Kaplan–Meier analysis of HCC development time of patients different risk score. (c)-(d) Univariate and multivariate analysis of the risk signature for HCC development time for early-stage liver cirrhosis patients. (e) AUC of the risk signature in predicting 2-year, 5-year, and 10-year HCC development for early-stage liver cirrhosis patients.

Having found 42 abnormally expressed genes, which may be served as biomarkers for the identification of early-stage liver cirrhosis patients who were at high risk for HCC, we then tried to comprehensively explore the association of these abnormally expressed genes with the development of HCC. First, 216 early-stage liver cirrhosis patients were randomly divided into training cohort (*N* = 108) and validation cohort (*N* = 108). Next, 14 genes were found to be associated with HCC development in patients of the training cohort by univariate Cox regression analysis (Supplementary Material 3). Then, multivariate Cox regression analysis was performed to further screen out the most HCC development-associated genes (including SEMA4D, RBM28, RPS3A, AGPAT1, COPS4, DPP3, NPLOC4, and YEATS2). Finally, a risk signature was constructed based on the coefficients weighted by multivariate Cox regression analysis. The risk score was calculated as follows: risk score = (−2.07*∗*SEMA4D expression) + (1.71*∗*RBM28 expression) + (0.83*∗*RPS3A expression) + (0.73*∗*AGPAT1 expression) − (1.23*∗*COPS4 expression) + (1.44*∗*DPP3 expression) + (0.32*∗*NPLOC4 expression) + (0.67*∗*YEATS2 expression). We calculated the risk score for each patient. Patients with a risk score > 4 were classified into high-risk group (*N* = 24), and others with a risk score < 4 were assigned to low-risk group (*N* = 84) by the X-Tile software (http://tissuearray.org/, version 3.6.1). Higher risk scores were found in liver cirrhosis patients who would develop HCC compared to those patients who would not develop HCC (*p* < 0.001, Figure 4(a)). Patients in the high-risk group had shorter HCC development time than that of the low-risk group (HR = 12.24, 95% CI: 5.8–25.86, *p* < 0.001, Figure 4(b)). Besides, univariate Cox regression analysis and multivariate Cox regression analysis also indicated that the risk signature was an independent prognostic factor for HCC development (HR = 11.02, 95% CI: 5.12–23.70, *p* < 0.001, Figures 4(c) and 4(d)). Moreover, the areas under ROC curve (AUC) of the risk signature for predicting 2-, 5-, and 10-year HCC development were 0.767, 0.909, and 0.859, respectively (Figure 4(e)), which showed good predictive value of the risk signature in predicting HCC development.

### 3.4. Validation of the Risk Signature in Patients of the Validation Cohort and the Whole Cohort

**Figure 5:**
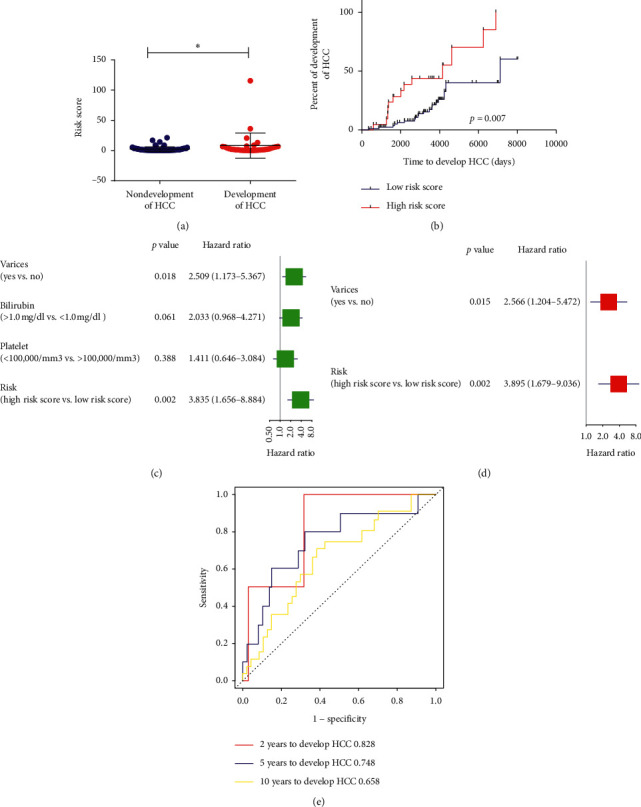
Validation of the risk signature in patients of the validation cohort. (a) Risk score between early-stage liver cirrhosis patients who would or would not develop HCC. (b) Kaplan–Meier analysis of HCC development time of patients different risk score. (c)-(d) Univariate and multivariate analysis of the risk signature for HCC development time for early-stage liver cirrhosis patients. (e) AUC of the risk signature in predicting 2-year, 5-year, and 10-year HCC development for early-stage liver cirrhosis patients.

**Figure 6:**
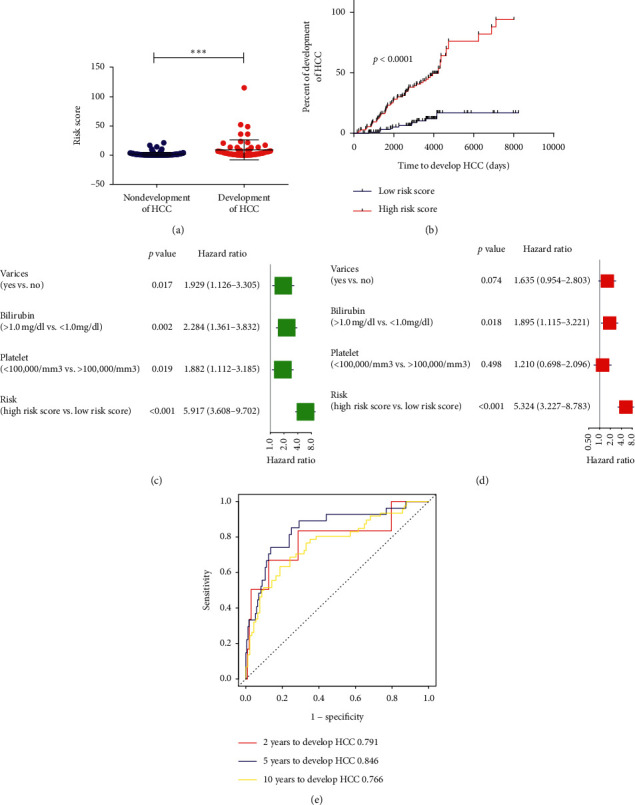
Validation of the risk signature in patients of the whole cohort. (a) Risk score between early-stage liver cirrhosis patients who would or would not develop HCC. (b) Kaplan–Meier analysis of HCC development time of patients different risk score. (c)-(d) Univariate and multivariate analysis of the risk signature for HCC development time for early-stage liver cirrhosis patients. (e) AUC of the risk signature in predicting 2-year, 5-year, and 10-year HCC development for early-stage liver cirrhosis patients.

To further test the applicability of the risk signature, we further examined its prognostic value in the validation cohort and the whole cohort. Similarly, risk score for each patient was also calculated, and then they were assigned into high-risk group (*N* = 22) and low-risk group (*N* = 86) in the validation cohort and high-risk group (*N* = 46) and low-risk group (*N* = 170) in the whole cohort with the same cut-off value. Likewise, higher risk scores were found in liver cirrhosis patients who would develop HCC compared to those patients who would not develop HCC in the validation cohort and the whole cohort (all *p* < 0.05, Figures 5(a) and 6(a)). Patients in the high-risk group had shorter HCC development time than that of the low-risk group in the validation cohort (HR = 3.84, 95% CI: 1.66–8.88, *p*=0.002, Figure 5(b)) and in the whole cohort (HR = 5.92, 95% CI: 3.61–9.70, *p* < 0.001, Figure 6(b)). Besides, univariate Cox regression analysis and multivariate Cox regression analysis also suggested that the risk signature was an independent prognostic factor for HCC development in the validation cohort (HR = 3.89, 95% CI: 1.68–9.04, *p*=0.002, Figures 5(c) and 5(d)) and in the whole cohort (HR = 5.32, 95% CI: 3.23–8.78, *p*=0.002, Figures 6(c) and 6(d)). Moreover, the AUC of the risk signature for predicting 2-, 5-, and 10-year HCC development were 0.828, 0.748, and 0.658 in the validation cohort (Figure 5(e)) and 0.791, 0.846, and 0.766 in the whole cohort (Figure 6(e)), which validated the good predictive value of the risk signature in predicting HCC development.

### 3.5. Association of the Risk Signature with the OS of Early-Stage Liver Cirrhosis Patients

**Figure 7:**
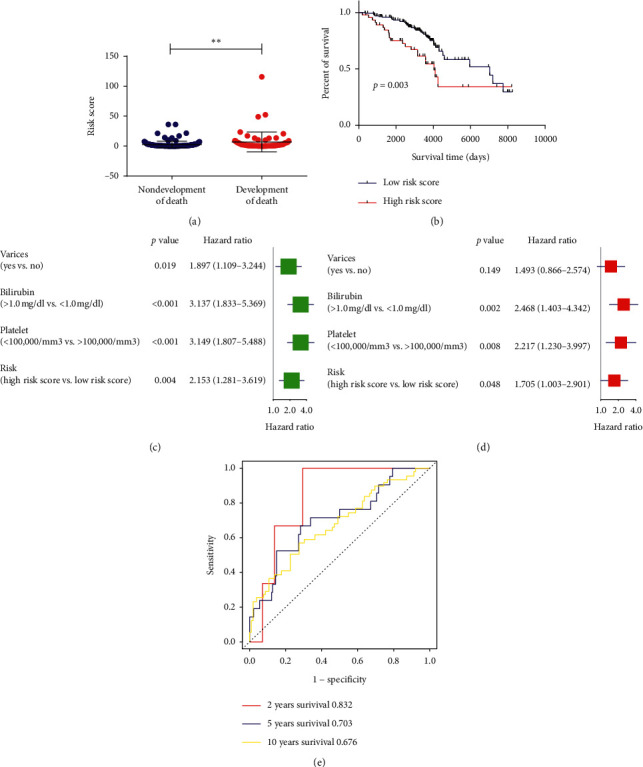
Association of the risk signature with the overall survival (OS) of early-stage liver cirrhosis patients. (a) Risk score between early-stage liver cirrhosis patients who would or would not develop death. (b) Kaplan–Meier analysis of OS time of patients different risk score. (c)-(d) Univariate and multivariate analysis of the risk signature for OS time for early-stage liver cirrhosis patients. (e) AUC of the risk signature in predicting 2-year, 5-year, and 10-year OS for early-stage liver cirrhosis patients.

After validation of the risk signature in the prediction of HCC development, we next further analyzed the association of the risk signature with the OS of early-stage liver cirrhosis patients in the whole cohort. Similarly, higher risk scores were found in liver cirrhosis patients who would develop death compared to liver cirrhosis patients who would not (*p* < 0.01, Figure 7(a)). Patients in the high-risk group had shorter OS time than that of the low-risk group (HR = 2.15, 95% CI: 1.28–3.62, *p*=0.003, Figure 7(b)). Besides, univariate Cox regression analysis and multivariate Cox regression analysis also indicated that the risk signature was an independent prognostic factor for OS (HR = 1.71, 95% CI: 1.003–2.91, *p*=0.048, Figures 7(c) and 7(d)). Moreover, the AUC of the risk signature for predicting 2-, 5-, and 10-year OS were 0.832, 0.703, and 0.676, respectively (Figure 7(e)), which also suggested good predictive value of the risk signature in predicting OS of early-stage liver cirrhosis patients.

### 3.6. Association of the Risk Signature with the Infiltrating Immune Cells of Early-Stage Liver Cirrhosis Patients

**Figure 8:**
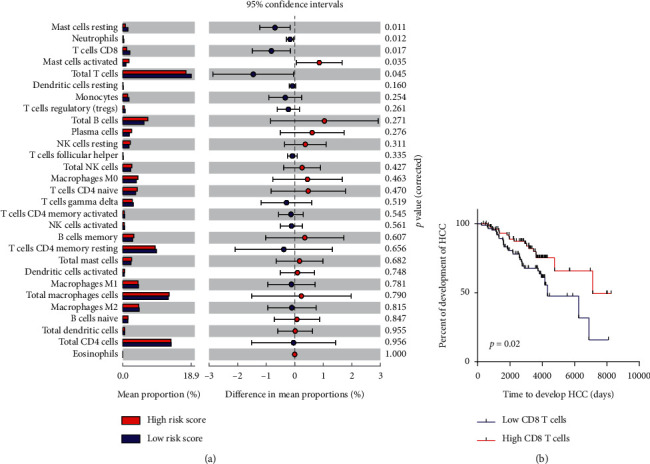
Association of the risk signature with the infiltrating immune cells of early-stage liver cirrhosis patients. (a) Landscape of tumor-infiltrating immune cells in early-stage liver cirrhosis patients of different risk score. (b) Kaplan–Meier analysis of HCC development time of patients with different levels of CD8 T cells.

Previous researches had showed that immune system played an important role in protecting against cancer development [19], so we next tried to analyze the relationship of the risk signature with the infiltrating immune cells of early-stage liver cirrhosis patients. We used CIBERSOR to calculate infiltrating immune cells in early-stage liver cirrhosis patients. As is shown in Figure 8(a), significant proportions of resting mast cells, neutrophils, CD8 T cells, and total T cells were found to be enriched in HCC patients with low risk score, while only activated mast cells were found to be enriched at patients with high risk score (all *p* < 0.05, Figure 8(a)). Further analysis showed that higher CD8 T cells were associated with longer HCC development time (HR = 1.96, 95% CI: 1.11–3.40, *p*=0.02, Figure 8(b)), which may indicate that the signature may affect HCC development of early-stage liver cirrhosis patients by the regulation of the infiltration of CD8 T cells.

### 3.7. Prognostic Value of the Risk Signature in HCC Patients from TCGA Cohort, GSE14520 Cohort, and ICGC Cohort

**Figure 9:**
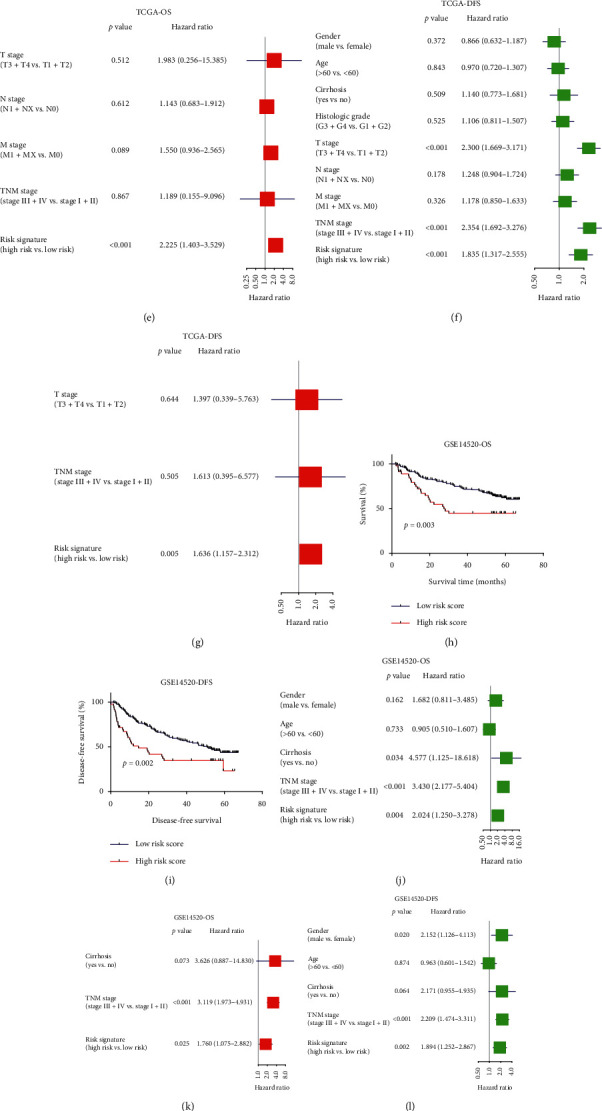
Prognostic value of the risk signature in HCC patients from TCGA cohort, GSE14520 cohort, and ICGC cohort. (a) Risk score between normal controls and HCC patients. (b)-(c) Kaplan–Meier analysis of OS and DFS time of patients with different risk score of TCGA cohort. (d)–(g) Univariate and multivariate analysis of risk signature for OS and DFS time of HCC patients of TCGA cohort. (h)-(i) Kaplan–Meier analysis of OS and DFS time of patients with different risk score of GSE14520 cohort. (j)–(m) Univariate and multivariate analysis of risk signature for OS and DFS time of HCC patients of GSE14520 cohort. (n) Kaplan–Meier analysis of OS time of patients with different risk score of ICGC cohort. (o)-(p) Univariate and multivariate analysis of risk signature for OS time of HCC patients of ICGC cohort.

Having found that the risk signature was significantly associated with HCC development and OS of early-stage liver cirrhosis patients, we then aimed to examine whether the risk signature was associated with the prognosis of HCC patients. Likewise, risk score for each HCC patients was calculated and then assigned into high-risk group and low-risk group by the X-Tile software. In TCGA cohort, higher risk scores were found in HCC patients compared to normal controls (*p* < 0.001, Figure 9(a)), indicating that the signature may also be used to serve as biomarker to distinguish HCC patients from healthy controls. Moreover, patients in the high risk group had shorter OS and DFS time than that of the low-risk group (OS : HR = 2.46, 95% CI: 1.60–3.79, *p* < 0.001; DFS : HR = 1.84, 95% CI: 1.32–2.56, *p* < 0.001, Figures 9(b), and 9(c)); univariate and multivariate Cox regression analysis also suggested that the risk signature was an independent prognostic factor for OS and DFS time for HCC patients (OS : HR = 2.23, 95% CI: 1.40–3.53, *p* < 0.001; DFS : HR = 1.64, 95% CI: 1.16–2.31, *p*=0.005, Figures 9(d)–9(g)). Similar results were found in GSE14520 cohort and ICGC cohort. In GSE14520 cohort, patients in the high-risk group had shorter OS and DFS time than that of the low-risk group (OS : HR = 2.20, 95% CI: 1.25–3.28, *P*=0.004; DFS : HR = 1.89, 95% CI: 1.25–2.87, *P*=0.002, Figures 9(h) and 9(i)); univariate and multivariate Cox regression analysis also suggested that the risk signature was an independent prognostic factor for OS and DFS time of HCC patients (OS : HR = 1.76, 95% CI: 1.08–2.88, *P*=0.05; DFS : HR = 1.83, 95% CI: 1.20–2.79, *P*=0.005, Figures 9(j)–9(m)). In ICGC cohort, patients in the high risk group had shorter OS than that of the low risk group (HR = 5.24, 95% CI: 2.87–9.57, *P* < 0.001Figure 9(n)); univariate and multivariate Cox regression analysis also suggested that the risk signature was an independent prognostic factor for OS and DFS time for HCC patients (HR = 5.33, 95% CI: 2.84–10.0, *P* < 0.001; Figures 9(o) and 9(p)). Taken together, the signature was also associated with the prognosis of HCC patients.

### 3.8. Identification of Bioactive Compounds as Chemopreventive Treatment for Prevention of HCC Development Based on CMap Analysis

We performed KEGG analysis to explore the underlying pathological pathways by which the signature used to influence the development of HCC for early-stage liver cirrhosis patients. As was shown in Supplementary Figure A, pathological pathways, such as “negative regulation of apoptotic process,” “positive regulation of NF-kappaB transcription factor activity,” “nucleotide-excision repair, preincision complex assembly,” “glycogen biosynthetic process,” “positive regulation of vascular endothelial growth factor receptor signaling pathway,” “regulation of angiogenesis,” “regulation of cyclin-dependent protein serine/threonine kinase activity,” and KEGG pathways, such as “chemical carcinogenesis,” “base excision repair,” “glucagon signaling pathway,” and “glycolysis/gluconeogenesis,” were significantly enriched in patients with high risk scores compared to patients with low risk scores, suggesting that the aforementioned pathological pathways played an important roles of the risk signature in influencing the development of HCC. With the help of the CMap dataset, four bioactive compounds including trichostatin A, vorinostat, valproic acid, and tinidazole were identified as potential chemopreventive compounds. Among all these four bioactive compounds, trichostatin A, vorinostat, and valproic acid were histone deacetylases (HDAC) inhibitors, which had been demonstrated to exhibit antitumor efficacy via activation of classic and alternative cell death molecular cascades [20, 21]. So, these bioactive compounds may prevent cirrhosis from development of HCC by targeting the pathological pathways that are mediated by the genes used for construction of the risk signature (Supplementary Figure B). Information of these bioactive compounds is shown in Table 3.

## 4. Discussion

Liver cirrhosis is highly related to hepatitis B virus infection, hepatitis C virus infection, nonalcoholic fatty liver disease (NAFLD), and alcoholic fatty liver disease and is the major driver of HCC [6]. The initial manifestation of HCC patients was often a cirrhotic liver. [7, 8] Further understanding of the molecular mechanism in the transformation of liver cirrhosis to HCC, especially early HCC, would be of great help for the identification of potential new biomarkers for HCC screening in cirrhosis patients. Previously, He et al. and Jiang et al. analyzed pivotal genes and pathways involved in the transformation of liver cirrhosis to HCC. They found that Hub genes, such as CDK1, RRM2, CDKN3, and KEGG pathways, such as cell cycle and p53 signaling pathways, were the key genes and KEGG pathways for the transformation of liver cirrhosis to early HCC [14, 15]. Different from their studies, in the present study, we mainly focused on the key genes and KEGG pathways for the transformation of liver cirrhosis to early HCC, which may provide other valuable information in understanding the molecular mechanism for the occurrence of HCC. We found that KEGG pathways, such as cell adhesion molecules (CAMs), PI3K-Akt signaling pathway, focal adhesion, antigen processing and presentation, chemokine signaling pathway, central carbon metabolism in cancer, and choline metabolism in cancer, play a pivotal role in the development of HCC [22–26]. For example, chemokine signaling pathway plays a central role in mediating inflammation and regeneration in chronic liver diseases. Inflammatory chemokines recruited innate and adaptive immune cells and thus promoted the composition of the local disease-specific microenvironment, which was the basis for the development of HCC. Besides, regulatory T cells and myeloid-derived suppressor cells could be recruited to liver by chemokine signaling pathway to exert their immunosuppressive effects by inhibiting NK and CD8+ T cells, and thus promoting the initiation and progression of liver cancer [25]. We also found that KEGG pathways, such as T cells differentiation (including Th1, Th2, and Th17 cells), B cells receptor signaling pathway, antigen processing and presentation, central carbon metabolism in cancer, and choline metabolism in cancer were the key pathways for the transformation of early HCC from cirrhosis, indicating that changes in cell immune and nutrient metabolism may be early events on the occurrence of HCC [27–29]. Moreover, we identified 42 genes, such as SEMA4D, RBM28, and RPS3A, whose expression had become abnormal in early-stage liver cirrhosis patients before HCC development, indicating that these 42 abnormally expressed genes may play an important role in the transformation of cirrhosis to early HCC and they may be served as biomarkers for the identification of early-stage liver cirrhosis patients who were at high risk for HCC development.

Although antiviral therapies could reduce the risk of HCC development in viral hepatitis patients, once liver cirrhosis is established, no available preventive strategies could eliminate the risk of HCC development [30–33]. Considering only 12% of new HCC patients could be diagnosed through current surveillance strategy [13], we hope to explore new effective prognostic signature to identify cirrhosis patients who are at high risk for HCC development. In order to address this problem, Hoshida et al. and King et al. developed a 186-gene signature for prognosis of cirrhosis patients of GSE15654, and they found the signature was an independent predictor of HCC development, but the risk for HCC development in high-risk patients was no more than 3.5 times than that in low-risk patients [34, 35]. Recently, Moeini et al. developed a risk signature for the prediction of HCC development in cirrhosis patients of GSE15654 on the basis of identifying genes that regulate immune response which could contribute to hepatocarcinogenesis. They found that the signature was an independent predictor of HCC occurrence. There was a 2.4-times risk of HCC development for high-risk patients compared to that in low-risk patients [36]. However, the signature developed for discrimination was with fair hazard ratios smaller than 3.5, and none of the them further calculated the ROC for the prediction of HCC development. Differently, in the present study, we developed and validated an 8-gene risk signature for the prediction of HCC development for early-stage cirrhosis patients on the basis of 42 abnormally expressed genes whose expression had been altered in early cirrhosis patient before HCC development. Among all these 8 genes, only the mechanism of RPS3A in the HCC had been explored. Lim et al. found that overexpressed RPS3A could promote the stability and functional activity of HBx protein by its chaperoning activity and thus promote HBx to exert effective viral oncogenic activity and contribute to HCC development [37]. Although there were no reports about the role of the other 7 genes in HCC, future characterization of them may provide new insights into the development and progression of HCC and discovery of potential novel therapeutic targets. With the help of this signature, liver cirrhosis patients could be divided into two distinct subgroups, and the risk for HCC development in high-risk patients was 5.42 times than that in low-risk patients. Besides, the risk signature was an independent prognostic factor for HCC development in cirrhosis patients. Moreover, the AUC of risk signature for predicting 2-, 5-, and 10-year HCC development were 0.791, 0.846, and 0.766, indicating good predictive value of the risk signature in predicting HCC development. Therefore, the risk signature may exhibit great underlying clinical implications for management of cirrhosis patients. In this regard, high-risk cirrhosis patients may need more intensive surveillance and even active chemopreventive treatment to reduce the occurrence of HCC and improve prognosis, while low-risk cirrhosis patients may receive less active follow-up and even can avoid the unnecessary adjuvant therapies.

Previous researches have showed that immune system played an important role in protecting against cancer development. In short, innate immune cells, such as macrophages, dendritic cells, and NK cells, could monitor and destroy external and internal pathogens and nascent tumor cells by directly and indirectly in conjunction with adaptive immune T cells and B cells [19]. In the present study, we also found that the risk signature was associated with infiltrating immune cells of cirrhosis patients. Higher proportions of resting mast cells, neutrophils, CD8 T cells, and total T cells were found to be enriched in HCC patients with low risk score, while only activated mast cells was found to be higher at patients with high risk score, which indicates the immune-enriched phenotype in low-risk patients and immune-depleted phenotype in high-risk patients. Moreover, further analysis found that higher CD8 T cells was associated with longer HCC development time, suggesting an important antitumorigenic role played by CD8 T cells in HCC development. It is well known that CD8 T cells could eradicate established tumors [19]. Broz et al. have found that CD103 + DC-mediated cross-presentation of tumor antigens could activate CD8 T cells to be cytotoxic CD8 T cells (CTLs), which could effectively control tumor outgrowth and mediate efficient tumor [38]. Similar to the results by Broz et al. and Garnelo et al., the degree of infiltrated T cells and B cells of tumor tissues is significantly associated with improved prognosis in HCC patients [28]. Moreover, Shalapour et al. also found that CTLs could actively prevent HCC occurrence in a mouse models of NASH-promoted HCC as unleashing CTL activity causes regression of established HCC while interference with activation of CTLs by IgA + cells promotes HCC development [39]. Therefore, the above studies may indicate that the risk signature may affect the HCC development of early-stage liver cirrhosis patients by regulation of the infiltration of CD8 T cells.

Up to date, there is still no established preventive treatment for cirrhosis patients who are at risk for HCC development [30]. Reducing the incidence and mortality of HCC patients requires not only the advances in development of curative treatment for early lesions, but also identifying cirrhosis patients who are at high risk for HCC development and development of chemopreventive. In this scenario, we developed and validated a risk signature for prediction of HCC development of cirrhosis patients. Besides, we also found that “negative regulation of apoptotic process,” “glycogen biosynthetic process,” “regulation of angiogenesis,” “regulation of cyclin-dependent protein serine/threonine kinase activity,” “glucagon signaling pathway,” and “glycolysis/gluconeogenesis” were found to be significantly enriched in patients with high risk scores, indicating that the aforementioned pathological pathways played an important role of the risk signature in influencing the development of HCC. On the basis of the risk signature, we also identified that trichostatin A, vorinostat, and valproic acid may be the promising potential bioactive compounds as novel chemopreventive treatment for the prevention of HCC development by targeting the genes used for construction of the risk signature. These bioactive compounds are all HDAC inhibitors and exhibit preclinical antitumor efficacy. HDAC inhibitors can affect various pathways and lead to transformed cell death. They can induce DNA damage and repair, modify gene expression, cause cell growth arrest, induce apoptosis, and act as antiangiogenic and antimetastatic factors [20, 21], and cell cycle (regulation of cyclin-dependent protein serine/threonine kinase activity), reduced apoptosis (negative regulation of apoptotic process), and angiogenesis (regulation of angiogenesis) were found to be significantly enriched in patients with high risk scores (Supplementary Figure A). For example, Zhou et al. have found that vorinostat can lead to HCC cell morphology changes, growth inhibition, cell cycle blockage, and apoptosis in vitro and suppressed the growth of subcutaneous HCC xenograft tumours in vivo via upregulation of p21^Waf1/Cip1^ and p19^INK4d^ [40]. Freeze et al. have shown that trichostatin A and valproic acid could not only inhibit the proliferation, clonogenicity, and migration of HCC cells, but also enhance the efficacy of sorafenib in killing sorafenib-susceptible cells and reestablished sorafenib sensitivity in resistant HCC cells [41]. It is proved that alterations in homeostasis of glucose play an important role in the development of tumors, loss of fructose-1, 6-bisphosphatase (FBP1), a rate-limiting enzyme in gluconeogenesis, was found to be oncogenic in various cancer cells including HCC and colon cancer cells [42], suggesting that modulation of gluconeogenesis also plays an equal role in tumorigenesis. Consistent with this, we also found “glycolysis/gluconeogenesis” to be significantly enriched in patients with high risk scores. Yang et al. have found that inhibition of histone deacetylases by HDAC inhibitors suppresses glucose metabolism and hepatocellular carcinoma growth by restoring FBP1 expression [43]. Taken together, HDAC inhibitors, such as trichostatin A, vorinostat, and valproic acid, may be exploited to be potential chemopreventive treatment for prevention of HCC development for cirrhosis patients. However, future clinical studies are still needed for further confirmation.

Compared with previous studies, our study has several strengths. First, the risk signature was developed on the basis of 42 DEGs identified between liver cirrhosis patients and early HCC patients. The expression of those genes had been altered in early-stage liver cirrhosis patients before HCC development, so the signature could effectively discriminate cirrhosis patients who were at high risk for HCC development from cirrhosis patients who were at low risk for HCC development with hazard ratios more than 5.0. It also showed good predictive value in predicting HCC development. Second, the prognostic and predictive value of the risk signature were validated in an internal cohort. Finally, the risk signature could also effectively stratify HCC patients into high-risk patients with shorter OS and DFS time and low-risk patients with longer OS and DFS time and it was an independent prognostic factor for OS and DFS time in HCC patients from three different HCC cohorts.

Although the risk signature exhibited good performance for the prediction of HCC development in cirrhosis patients, some limitations should be addressed. First, some basic parameters of the cirrhosis patients from GSE15654, such as age, gender, especially AFP level were missing, so we could not further perform a comparative analysis of predictive value between the risk signature and AFP. Second, limited information was provided to explore the relation between the risk signature and regulation of CD8 T cells. Third, we did not validate the prognostic value of the risk signature in an external cohort, especially in prospective cohorts with larger sample sizes. Finally, we also did not validate chemopreventive efficacy of trichostatin A, vorinostat, and valproic acid for prevention of HCC development in vivo and in vitro experiment.

In conclusion, we developed and validated an 8-gene risk signature for prediction of HCC development, which showed good performance in the discrimination ability and predictive ability for cirrhosis patients. The risk signature may be a useful tool to set up more individualized follow-up interval schedules and HDAC inhibitors, such as trichostatin A, vorinostat, and valproic acid, may be exploited to be a potential chemopreventive treatment for the prevention of HCC development for cirrhosis patients.

## Figures and Tables

**Table 1:** Basic characteristics of 216 patients with early-stage liver cirrhosis from GSE15654.

Variables	Liver cirrhosis patients (*N* = 216)
Varices (yes/no/NA)	52/159/5
Bilirubin (<1.0 mg/dl/ ≥1.0 mg/dl)	108/108
Platelet (<100,000/mm^3^/≥100,000/mm^3^)	99/117
Development of HCC (yes/no)	65/151
Times to develop HCC (days, median)	3230 (175–8256)
Development of death (yes/no)	66/150
Times to death (days, median)	3580.5 (194–8256)

**Table 2:** Basic characteristics of HCC patients from TCGA, GSE14520, and ICGC HCC cohorts.

Variables	TCGA cohort (*N* = 377)	GSE14520 cohort (*N* = 220)	ICGC cohort (*N* = 232)
Gender (male/female)	255/122	190/30	171/61
Age (years, ≤60/>60/NA)	180/196/1	181/39	50/182
Cirrhosis (yes/no/NA)	81/137/159	202/18	NA
Histologic grade (*G*1/*G*2/*G*3/*G*4/NA)	55/180/124/13/5	NA	NA
*T* stage (I/II/III/IV/TX/NA)	185/95/81/13/1/2	NA	NA
*N* stage (*N*0/*N*1 + NX/NA)	257/119/1	NA	NA
*M* stage (*M*0/*M*1 + MX)	272/105	NA	189/43
TNM stage (I/II/III/IV/NA)	175/87/86/5/24	93/77/48/-/2	36/106/71/76

**Table 3:** Bioactive compounds identified as a potential chemopreventive treatment for the prevention of HCC development based on CMap analysis.

Drug name	Dose	Cell line	Score	Instance ID
Trichostatin A	100 nM	PC3	−0.877	4184
Vorinostat	10 *µ*M	MCF7	−0.862	1645
Trichostatin A	100 nM	PC3	−0.856	1212
Trichostatin A	100 nM	HL60	−0.847	1561
Trichostatin A	100 nM	MCF7	−0.835	4237
Trichostatin A	100 nM	PC3	−0.826	4237
Valproic acid	1 mM	HL60	−0.821	1150
Tinidazole	16 *µ*M	MCF7	−0.820	3430
Trichostatin A	100 nM	HL60	−0.809	1612
Trichostatin A	100 nM	PC3	−0.800	6316

## Data Availability

The data of this study are available from the corresponding web page link, including GDC data portal (https://cancergenome.nih.gov/), ICGC portal (https://dcc.icgc.org/projects/LIRI-JP), and GEO database (https://www.ncbi.nlm.nih.gov/geo/).

## Abbreviations

HCC: Hepatocellular carcinoma
DEGs: Differentially expressed genes
TCGA: The Cancer Genome Atlas
GEO: Gene expression omnibus
ICGC: International Cancer Genome Consortium
OS: Overall survival
DFS: Disease-free status
AUC: Areas under ROC curve
HDAC: Histone deacetylases
NAFLD: Nonalcoholic fatty liver disease
CTLs: Cytotoxic CD8 T cells
FBP1: Fructose-1,6-bisphosphatase.

## Supplementary Materials

Supplementary Materials
*Supplementary Figure A*. The underlying pathological pathways by which the signature used to influence the development of HCC for early-stage liver cirrhosis patients. *Supplementary Figure B*. Schematic depicting the pathological pathways mediated by signature and inhibited by suggested bioactive compounds (HDAC inhibitors). *Supplementary Material 1*. DEGs between early HCC patients and cirrhosis patients identified by overlapping GSE63898 and GSE89377 datasets. *Supplementary Material 2*. DEGs identified by overlapping GSE63898, GSE89377, and GSE15654 datasets. *Supplementary Material 3*. HCC development related genes identified by univariate analysis in the patients of the training cohort.
