# Supplementary material for: Identification of New Biomarker for Prediction of Hepatocellular Carcinoma Development in Early-Stage Cirrhosis Patients
Source: J Oncol. 2021 Jul 20;2021:9949492. doi: 10.1155/2021/9949492 (PMC8318773; doi:10.1155/2021/9949492)
Supplement: Supplementary Materials — Supplementary Figure A. The underlying pathological pathways by which the signature used to influence the development of HCC for early-stage liver cirrhosis patients. Supplementary Figure B. Schematic depicting the pathological pathways mediated by signature and inhibited by suggested bioactive compounds (HDAC inhibitors). Supplementary Material 1. DEGs between early HCC patients and cirrhosis patients identified by overlapping GSE63898 and GSE89377 datasets. Supplementary Material 2. DEGs identified by overlapping GSE63898, GSE89377, and GSE15654 datasets. Supplementary Material 3. HCC development related genes identified by univariate analysis in the patients of the training cohort. [file 9949492.f1.zip › 9949492.f1/Supplementary Material 1.pdf]

DEGs between early HCC patients and cirrhosis patients identifed by overlapping GSE63898 and GSE89377 datatset

Gene	logFC of GSE63898	adj.pValue	logFC of GSE89377	adj.pValue
CXCL12	-0.672442177	1.95364E-52	-0.275096249	0.001292825
CXCL14	-0.610412807	3.93283E-61	-0.231853038	0.026826115
DPT	-0.597365317	7.89283E-49	-0.381975097	0.003878474
C7	-0.570249198	1.64777E-44	-0.260292449	0.000646412
RND3	-0.548334136	5.11894E-52	-0.072550755	0.036522301
IGFBP3	-0.542300989	8.27746E-45	-0.227484989	0.00937298
DCN	-0.53495774	7.9739E-50	-0.282179644	0.000646412
TACSTD2	-0.528495384	1.33396E-54	-0.436954416	0.002262443
LUM	-0.524078082	3.4368E-26	-0.187385483	0.003878474
CCL2	-0.519912582	5.1523E-39	-0.201600002	0.006140918
PTGDS	-0.491117442	2.6803E-27	-0.331053215	0.002262443
SFRP5	-0.489685024	9.62421E-53	-0.354948495	0.001292825
OLFML3	-0.47523197	1.65947E-52	-0.24716737	0.000323206
SPINT2	-0.472912868	1.20777E-41	-0.378947651	0.006140918
CYP39A1	-0.468723266	8.03327E-37	-0.156632109	0.019392372
LAMA2	-0.451459644	1.68981E-29	-0.192958109	0.006140918
S100A8	-0.449423778	2.44805E-32	-0.154903041	0.026826115
ANK3	-0.417826072	1.07764E-43	-0.080592982	0.006140918
KRT7	-0.414748656	4.26477E-27	-0.24617858	0.013188555
FBLN5	-0.414538253	9.94406E-32	-0.239478278	0.000646412
DNASE1L3	-0.391409688	1.19054E-50	-0.091671339	0.048480931
IL7R	-0.385541077	1.0294E-29	-0.266269287	0.006140918
ADAMTS1	-0.382339155	1.85635E-43	-0.283362123	0.009763047
CCL21	-0.374969219	6.71218E-28	-0.292219525	0.036522301
ANXA3	-0.371504014	3.55925E-46	-0.088904633	0.000646412
AGTR1	-0.369063993	1.98352E-36	-0.085415365	0.048480931
CCDC3	-0.367070677	9.99115E-41	-0.272535609	0.001292825
PLAC8	-0.366357	7.22903E-57	-0.188076024	0.036522301
CHST4	-0.362661001	2.38792E-54	-0.505049324	0.000646412
LYVE1	-0.360791636	1.22277E-50	-0.18101254	0.001292825
PCDH9	-0.358180741	8.17439E-45	-0.032775736	0.003878474
CYR61	-0.357031277	6.51864E-38	-0.221708715	0.001292825
MXRA5	-0.357006984	7.70924E-36	-0.13496515	0.013574661
MMP7	-0.353268633	1.69791E-27	-0.504650794	0.001292825
DBH	-0.343541244	9.42612E-55	-0.234696247	0.019392372
LXN	-0.342190472	2.52902E-23	-0.279761478	0.003878474
CLDN10	-0.342014055	2.05038E-48	-0.320740563	0.003878474
MS4A6A	-0.341360677	2.52752E-42	-0.145442934	0.000323206
ANGPTL1	-0.330732985	3.26335E-42	-0.058754286	0.000323206
SPG20	-0.329901877	1.66999E-42	-0.0791023	0.001292825
PRELP	-0.325702312	1.15945E-26	-0.04761558	0.00937298
ARHGAP15	-0.32402606	1.07546E-30	-0.218649831	0.000646412
GNA14	-0.315701973	1.86702E-44	-0.075352943	0.00937298
IGFBP6	-0.313811845	6.95854E-32	-0.311459117	0.000646412
ITK	-0.313021548	1.0818E-22	-0.154360284	0.003878474
QSOX1	-0.3078201	1.98961E-34	-0.156676715	0.00937298
SULF2	-0.30585447	1.59046E-29	-0.132537294	0.006140918
SAMSN1	-0.30467653	1.17799E-35	-0.085330521	0.006140918
SRGN	-0.301603568	5.3261E-31	-0.148253213	0.003878474
CD53	-0.301242248	1.47559E-24	-0.105887011	0.000646412
IRF8	-0.300564668	1.07332E-52	-0.115896591	0.006140918
BASP1	-0.300337404	3.22327E-32	-0.130819248	0.002262443

PDGFRA	-0.29793246	5.68039E-45	-0.296439866	0.000646412
CHST9	-0.293073932	3.2695E-19	-0.192347477	0.001292825
MGC29506	-0.292848863	3.25331E-27	-0.31433741	0.006140918
PTPRC	-0.292129566	8.65913E-28	-0.057059362	0.006140918
CCL5	-0.290647324	6.15204E-17	-0.181679159	0.002262443
SH3YL1	-0.289565047	2.26563E-45	-0.252878136	0.001292825
EDNRB	-0.28780563	2.10038E-45	-0.103007488	0.000646412
ITGA9	-0.28668175	7.25331E-46	-0.131323128	0.003878474
PTN	-0.285198716	2.9818E-27	-0.082129324	0.000646412
STEAP4	-0.28053283	8.06158E-36	-0.16247447	0.000323206
AEBP1	-0.273011802	7.76146E-20	-0.222297768	0.002262443
ID3	-0.27293908	1.37699E-30	-0.175684748	0.006140918
CD52	-0.26936644	1.59145E-15	-0.147036628	0.013574661
EVI2B	-0.264528599	1.3239E-25	-0.134662443	0.000646412
LCP2	-0.26383453	8.24822E-29	-0.036825453	0.019392372
AXL	-0.26339692	2.44523E-38	-0.17810867	0.001292825
IL33	-0.25981017	1.18187E-26	-0.075594807	0.019392372
TSPYL5	-0.259469984	6.09381E-47	-0.173389467	0.001292825
KCNJ16	-0.253793084	4.87235E-42	-0.146594744	0.000323206
ZFPM2	-0.251582455	6.35328E-43	-0.110926227	0.006140918
PLA2G5	-0.244964324	1.71608E-32	-0.147591508	0.006140918
VSIG4	-0.244422324	5.60922E-31	-0.125885685	0.003878474
MTHFD2	-0.243536799	5.51085E-21	-0.116220704	0.003878474
MS4A7	-0.242382656	9.57214E-27	-0.145229808	0.001292825
TRIM22	-0.242132476	8.36204E-35	-0.110738373	0.003878474
TMEM154	-0.239805513	2.09844E-36	-0.152433102	0.026826115
LAMC3	-0.238043529	2.0863E-26	-0.274422462	0.002262443
PLSCR1	-0.234803002	3.62202E-38	-0.077415267	0.026826115
GGT5	-0.232840819	8.25329E-41	-0.182805175	0.002262443
PRNP	-0.232163218	2.60249E-33	-0.113542885	0.000646412
IFI16	-0.231649452	8.95037E-27	-0.151366622	0.00937298
FGFR2	-0.229539075	5.81529E-29	-0.058663058	0.026826115
FYN	-0.229222796	1.60267E-33	-0.065297253	0.048480931
CTGF	-0.22890824	5.94465E-16	-0.188052865	0.002262443
PROM1	-0.226748417	4.28925E-43	-0.428804111	0.000323206
GUCY1A3	-0.226210443	1.4256E-21	-0.181443775	0.006140918
HLA-DQA1	-0.225202113	6.1522E-25	-0.135310419	0.026826115
SPRY2	-0.224090204	1.17895E-41	-0.089608601	0.019392372
RGS1	-0.224039495	2.75368E-18	-0.185731919	0.00937298
PMP22	-0.223068155	2.76283E-14	-0.145450507	0.002262443
FXYD2	-0.222087029	8.49892E-25	-0.322693264	0.000646412
SLC25A36	-0.220446502	2.43447E-28	-0.123717748	0.000323206
PDE7B	-0.218000199	5.60947E-45	-0.084266498	0.013574661
TBXA2R	-0.213069986	4.3453E-56	-0.039118816	0.019392372
ENDOD1	-0.211683288	1.02697E-28	-0.171679551	0.006140918
ZEB2	-0.211490615	4.12835E-35	-0.122500938	0.001292825
LRRN3	-0.211313232	5.3152E-44	-0.088671642	0.002262443
ANXA1	-0.208103852	1.43538E-20	-0.166435462	0.00937298
GMFG	-0.208079396	4.77975E-20	-0.133958635	0.000646412
CD97	-0.206597226	6.08192E-22	-0.114282654	0.003878474
SYTL2	-0.206379375	2.16243E-22	-0.076770094	0.002262443
SYTL3	-0.205653891	4.9534E-31	-0.074862718	0.036522301
COX7A1	-0.205278991	2.42102E-21	-0.228826357	0.003878474
BTG2	-0.205258555	4.69997E-24	-0.166952947	0.048480931

PDE2A	-0.204721572	2.73755E-45	-0.079979934	0.003878474
MYO10	-0.203612639	2.61909E-51	-0.084956768	0.001292825
RAC2	-0.203306057	1.25852E-16	-0.161077808	0.006140918
SELP	-0.203236609	8.55966E-34	-0.050641289	0.00937298
TUBB6	-0.200511819	1.40883E-29	-0.11536845	0.003878474
RAI2	-0.199451117	1.538E-25	-0.054287391	0.002262443
DOCK10	-0.198933892	6.23681E-29	-0.113800002	0.006140918
AIF1	-0.198822638	8.00509E-29	-0.13792254	0.000323206
GSTP1	-0.198164795	1.10506E-29	-0.21773079	0.002262443
KCTD12	-0.197867843	8.28705E-20	-0.104526666	0.003878474
AOAH	-0.196754795	1.51325E-28	-0.12109778	0.002262443
FCRL3	-0.19659739	1.87138E-27	-0.038790714	0.036522301
SLFN11	-0.196171719	3.92914E-21	-0.124420478	0.019392372
TMEM100	-0.196144311	2.77006E-20	-0.158802907	0.001292825
EIF4E3	-0.192144811	5.90728E-31	-0.069573766	0.048480931
ANXA8	-0.191914829	8.94959E-44	-0.179967862	0.001292825
43891	-0.191880334	6.26701E-25	-0.0672524	0.000646412
ETS1	-0.19123178	2.33947E-16	-0.067262028	0.013574661
HCLS1	-0.188608181	1.86301E-31	-0.174507175	0.000646412
LDHB	-0.188561243	8.82449E-19	-0.164183729	0.001292825
PFKFB3	-0.187587857	7.44661E-42	-0.102324939	0.036522301
RUNX3	-0.187179902	7.74199E-35	-0.079179212	0.00937298
SLC5A1	-0.186956951	3.04594E-38	-0.216332672	0.006140918
SLC9A9	-0.185785454	6.02823E-36	-0.110712723	0.000646412
GRASP	-0.184700573	4.85001E-33	-0.08461434	0.026826115
PXDN	-0.18316195	6.87506E-24	-0.099045047	0.003878474
RBP1	-0.18260935	6.4287E-23	-0.310904625	0.000646412
SERPINB9	-0.180736021	2.52881E-40	-0.035874066	0.036522301
NAP1L5	-0.180469736	1.82701E-21	-0.065041335	0.002262443
NEXN	-0.178119694	2.14137E-18	-0.165393613	0.001292825
ABI3BP	-0.177988025	4.02775E-24	-0.159184419	0.000646412
GIMAP4	-0.177828347	4.43764E-16	-0.1137047	0.000646412
PLEK	-0.17728995	1.87296E-18	-0.089325071	0.00937298
CLEC10A	-0.175990283	1.00672E-28	-0.113181303	0.002262443
TAGLN	-0.175949743	1.18535E-12	-0.172886468	0.002262443
P2RY10	-0.175815289	1.97326E-16	-0.080239368	0.013574661
S100A9	-0.174850781	2.4666E-11	-0.122543431	0.003878474
FCER1G	-0.174101106	1.0681E-13	-0.124904861	0.002262443
IFITM1	-0.172958553	6.11411E-19	-0.134451183	0.006140918
HLA-DPB1	-0.172838551	8.81811E-25	-0.238083154	0.001292825
SYK	-0.172339518	4.72325E-18	-0.129409456	0.003878474
PMEPA1	-0.170397569	2.83982E-14	-0.130256433	0.000646412
ANXA13	-0.170316878	1.80922E-11	-0.172086027	0.048480931
IRAK3	-0.16902257	6.6446E-27	-0.097773335	0.019392372
TLR4	-0.167672045	8.99663E-37	-0.066923324	0.00937298
PLAGL1	-0.167022988	4.31276E-21	-0.046230222	0.000323206
LGALS3BP	-0.166682556	1.23643E-08	-0.151014395	0.007154102
ADAM28	-0.16593167	8.30554E-23	-0.039264715	0.00937298
ENG	-0.165923492	1.78397E-22	-0.117414929	0.003878474
LST1	-0.165673093	1.72644E-30	-0.101976957	0.003878474
F3	-0.165335503	1.82176E-24	-0.16386096	0.000323206
CORO1A	-0.165003234	4.50312E-16	-0.130381746	0.00937298
TNFRSF17	-0.16476738	2.59765E-23	-0.182929852	0.019392372
MMRN1	-0.16423822	2.05419E-20	-0.213716346	0.000323206

HLA-DPA1	-0.163459929	1.5375E-18	-0.132895196	0.013574661
PFKP	-0.161958356	2.95713E-11	-0.152235181	0.019392372
LHFP	-0.161833911	1.47353E-14	-0.215381169	0.001292825
ITGB2	-0.159809075	2.34908E-12	-0.108662435	0.002262443
LCP1	-0.159138718	7.08029E-16	-0.083495736	0.00937298
HK3	-0.158119637	8.19651E-34	-0.064793007	0.036522301
ARL4C	-0.157959593	1.47529E-21	-0.07204737	0.026826115
TSC22D3	-0.156110261	1.5904E-14	-0.109257409	0.013574661
C1QA	-0.154469151	9.21181E-14	-0.139501096	0.002262443
STMN2	-0.15413709	1.26394E-15	-0.208910842	0.036522301
LEPREL1	-0.153227468	2.48771E-44	-0.143799103	0.000323206
HHIP	-0.153156635	5.75219E-45	-0.035490503	0.048480931
MS4A4A	-0.152747527	8.03066E-15	-0.046436039	0.00937298
OLFM1	-0.1524344	6.34425E-41	-0.113579857	0.000323206
DPYSL3	-0.151471072	8.14404E-15	-0.184488847	0.026826115
GLT8D2	-0.151283948	1.86617E-23	-0.149712178	0.000646412
FZD1	-0.149889753	9.14361E-32	-0.047354455	0.002262443
EMP3	-0.149812909	1.27154E-18	-0.12135522	0.00937298
ADCY3	-0.149803837	5.06625E-18	-0.175002821	0.000646412
TAGAP	-0.149037429	4.32427E-39	-0.053895058	0.026826115
DSE	-0.148359344	2.24508E-35	-0.074195285	0.001292825
MYH10	-0.147837304	3.11633E-28	-0.150179548	0.000646412
LAPTM5	-0.146463288	1.23787E-11	-0.128365273	0.003878474
METRNL	-0.145719572	3.95523E-24	-0.099687174	0.00937298
CASP1	-0.145665077	1.2467E-19	-0.079919991	0.003878474
BANK1	-0.145190932	1.2467E-19	-0.079163965	0.00937298
SSPN	-0.14497866	2.75939E-27	-0.169708506	0.001292825
BTK	-0.144942898	7.08521E-23	-0.094359016	0.000323206
PPP1R16B	-0.144493057	3.98306E-23	-0.093161768	0.019392372
FAM129A	-0.142533534	3.12862E-09	-0.166898694	0.006140918
ATP1B3	-0.142481393	2.08897E-10	-0.096684097	0.013574661
IGFBP7	-0.141778988	7.83077E-19	-0.161790629	0.006140918
GYPC	-0.141599664	2.35841E-14	-0.127343673	0.001292825
LTBP4	-0.141143974	2.27013E-35	-0.123280775	0.013574661
CLEC4E	-0.140807953	4.98073E-20	-0.03516728	0.048480931
TNFAIP8	-0.139929113	4.28982E-15	-0.049820038	0.019392372
TYROBP	-0.139568225	2.03162E-17	-0.118591637	0.000646412
CYBA	-0.139560077	1.00899E-12	-0.182303199	0.000323206
ANK2	-0.139171041	9.09906E-37	-0.039065678	0.019392372
JAK2	-0.13904721	7.70692E-19	-0.054504767	0.00937298
S100A6	-0.139029723	4.92511E-11	-0.250227905	0.003878474
DOCK8	-0.138974629	1.26857E-37	-0.129780341	0.000646412
WSB1	-0.137436347	2.33991E-21	-0.047759065	0.013574661
PDLIM3	-0.136723035	1.11399E-08	-0.108243813	0.048480931
FZD8	-0.136183813	6.89207E-29	-0.072502643	0.002262443
RAB31	-0.13580198	2.8247E-13	-0.112028555	0.001292825
ZNF462	-0.135692889	5.86668E-26	-0.162207224	0.003878474
TBC1D10C	-0.134344763	2.14314E-15	-0.145485579	0.001292825
IQGAP1	-0.134333305	8.55363E-14	-0.062903497	0.006140918
APCDD1	-0.134246653	7.03938E-27	-0.221384487	0.002262443
IKZF1	-0.133214083	8.75454E-19	-0.178177265	0.013574661
CYBB	-0.133038676	8.13426E-18	-0.148890586	0.002262443
PPAP2B	-0.132773764	1.51227E-45	-0.057920818	0.048480931
MGP	-0.132734662	4.96937E-07	-0.224338747	0.019392372

EFEMP2	-0.132665282	3.77607E-15	-0.201718353	0.002262443
NDN	-0.132100709	5.56688E-12	-0.277246159	0.002262443
CD9	-0.131740167	6.88065E-12	-0.039643313	0.048480931
PIWIL4	-0.130868981	2.24228E-21	-0.054634929	0.019392372
TMEM159	-0.130327336	9.61558E-12	-0.070893458	0.000323206
LDB2	-0.130310748	1.01723E-16	-0.066074922	0.00937298
CCDC109B	-0.130178688	2.71798E-12	-0.174117634	0.001292825
PRSS23	-0.1301202	2.55486E-07	-0.113734871	0.00937298
TSHZ3	-0.130107342	4.45088E-24	-0.149990246	0.002262443
AQP1	-0.127892151	8.44439E-12	-0.138230931	0.00937298
LILRB5	-0.12767319	1.90275E-43	-0.08704648	0.006140918
PTPRE	-0.126806288	5.38834E-22	-0.076502001	0.002262443
MLKL	-0.126139264	3.37515E-13	-0.040534667	0.019392372
GLIS3	-0.125607167	1.59608E-13	-0.057927531	0.00937298
ABR	-0.125341411	4.33233E-19	-0.048937381	0.006140918
COL16A1	-0.12508862	2.65242E-11	-0.156334556	0.000646412
SH3BGRL	-0.124977176	5.46354E-12	-0.081144724	0.000323206
CPVL	-0.123964225	7.44736E-07	-0.170059082	0.000646412
PTPN22	-0.123779843	1.98378E-27	-0.032008931	0.013574661
HLA-DMB	-0.123652706	1.25579E-14	-0.102396623	0.019392372
PLEKHA2	-0.122815514	2.80923E-32	-0.05329703	0.000646412
GIMAP7	-0.122754919	3.6072E-11	-0.08492204	0.000646412
WIPF1	-0.122344075	3.04076E-19	-0.041166531	0.003878474
BGN	-0.122223725	2.58114E-32	-0.106885509	0.036522301
CD81	-0.121967375	1.2478E-29	-0.031743196	0.009718219
BNIP3L	-0.121112746	1.99886E-22	-0.098528707	0.000323206
CHSY1	-0.120764519	9.67378E-16	-0.111035158	0.026826115
HLA-DMA	-0.120536825	1.44569E-07	-0.103972297	0.048480931
TNFSF13B	-0.120227015	1.49302E-13	-0.116069707	0.013574661
TNFRSF21	-0.118822731	6.65333E-09	-0.156366465	0.036522301
ELF1	-0.118422404	3.78023E-29	-0.05832392	0.026826115
PNMA2	-0.118391096	1.39814E-15	-0.115386918	0.000646412
SEPW1	-0.117752075	4.39124E-09	-0.11691589	0.006140918
LRRC4C	-0.11713809	8.93273E-24	-0.078587336	0.006140918
RCBTB2	-0.116965404	7.08913E-35	-0.037716432	0.026826115
PODN	-0.116540766	6.19576E-30	-0.260983864	0.001292825
FAM129B	-0.116420089	1.4255E-11	-0.125257425	0.000646412
GCA	-0.116180897	7.61428E-16	-0.049600795	0.048480931
CXCL1	-0.115507954	9.73359E-23	-0.094118201	0.048480931
GPSM3	-0.115223001	1.02013E-21	-0.149503006	0.000323206
RASSF2	-0.113782476	5.43366E-24	-0.061108634	0.001292825
PDE8B	-0.113350399	1.73774E-22	-0.091879442	0.002262443
ECM1	-0.113217118	2.99913E-44	-0.143137543	0.00937298
SLC12A2	-0.113209352	6.65868E-08	-0.169108968	0.003878474
SLC4A4	-0.113172335	1.77248E-15	-0.031900383	0.048480931
CD24	-0.112423875	0.022614001	-0.253331579	0.026826115
CCDC146	-0.112011494	9.74408E-16	-0.146425689	0.00937298
DDR2	-0.111782774	8.00383E-17	-0.060635221	0.006140918
CTSK	-0.111638043	2.43787E-09	-0.079965396	0.019392372
ZSCAN18	-0.110989256	1.25761E-23	-0.207064815	0.048480931
RECK	-0.109843147	1.4838E-14	-0.093872149	0.003878474
GLIPR1	-0.109551231	4.92799E-08	-0.173458725	0.000646412
FAIM3	-0.108332908	7.25328E-30	-0.172138073	0.002262443
ANGPTL2	-0.108200533	7.91883E-15	-0.192949043	0.003878474

LPXN	-0.108128707	6.87372E-25	-0.069315103	0.030601606
ALDH1A3	-0.107777681	8.49309E-20	-0.117176897	0.048480931
HDGFRP3	-0.107603597	5.18471E-10	-0.0454994	0.006140918
RASGRP1	-0.10739159	1.93015E-22	-0.074358779	0.006140918
CSF3R	-0.106380547	1.92536E-34	-0.088422937	0.002262443
KCNK17	-0.106093871	6.60213E-32	-0.168519547	0.002262443
AKAP7	-0.105764015	6.74166E-36	-0.095708025	0.001292825
HEPH	-0.105525107	2.46367E-16	-0.050832714	0.00937298
HBEGF	-0.104924608	6.42701E-20	-0.110227352	0.036522301
PTPRN2	-0.104188826	1.13442E-28	-0.085310624	0.003878474
NAPSB	-0.104122872	3.42975E-30	-0.160188657	0.019392372
SPIRE1	-0.104087457	7.93699E-12	-0.178001329	0.002262443
FOLR2	-0.104008018	1.12068E-10	-0.145056672	0.002262443
IPPK	-0.103677367	1.43554E-24	-0.051396621	0.013574661
LBH	-0.102945615	2.42217E-11	-0.072482596	0.001292825
LGMN	-0.10291355	6.17732E-09	-0.059043228	0.003878474
FMOD	-0.10281403	2.00469E-23	-0.177251435	0.000646412
BAI3	-0.102465604	5.29587E-22	-0.135079706	0.000323206
MAL	-0.102089405	1.53022E-17	-0.11768338	0.000646412
ADD3	-0.100520462	2.60221E-09	-0.105913549	0.001292825
GIMAP8	-0.09985067	6.79671E-13	-0.058952876	0.013574661
TRPV6	-0.099506098	1.40883E-29	-0.073885336	0.000323206
TPM4	-0.099473713	8.73442E-05	-0.078972504	0.019392372
MYADM	-0.099093037	6.97081E-31	-0.09206466	0.036522301
AKR1B1	-0.098930385	1.69775E-15	-0.118827189	0.001292825
EOMES	-0.098616221	1.52669E-21	-0.089081249	0.048480931
FSTL1	-0.098586596	1.41842E-15	-0.095824732	0.026826115
SFT2D1	-0.098305443	9.30282E-15	-0.105561317	0.001292825
C1QTNF1	-0.098152936	8.52825E-23	-0.128641767	0.002262443
TMEM156	-0.097339678	4.54606E-19	-0.069092925	0.000646412
FUT8	-0.097123858	8.61352E-15	-0.044457855	0.019392372
MMP2	-0.096662794	4.11136E-15	-0.032800133	0.019392372
WDR66	-0.096465299	2.13334E-25	-0.086620096	0.003878474
FERMT3	-0.096287101	8.69632E-13	-0.090690002	0.003878474
EBI3	-0.096120809	6.48E-20	-0.140376181	0.001292825
S100A4	-0.095894276	2.22671E-06	-0.176038122	0.006140918
ARHGDIB	-0.095690887	4.05713E-13	-0.105023664	0.002262443
GABRB3	-0.095635617	9.30059E-27	-0.080004021	0.001292825
TUSC3	-0.095095563	3.05751E-13	-0.152696262	0.001292825
PLAT	-0.093665307	1.53092E-09	-0.156100513	0.002262443
CRYAB	-0.09350757	2.4095E-12	-0.23832271	0.019392372
TEK	-0.093322804	2.25093E-20	-0.089519574	0.013574661
LPAR1	-0.093008757	1.56845E-33	-0.109910646	0.026826115
LAX1	-0.092501659	4.10457E-18	-0.116918729	0.019392372
ANXA4	-0.092496319	1.48414E-20	-0.117456437	0.019392372
CD74	-0.091245421	9.06045E-08	-0.122907422	0.013574661
EPB41L3	-0.090902595	3.49226E-22	-0.094660692	0.00937298
LAMC2	-0.090360846	6.29716E-26	-0.065012463	0.019392372
S100A13	-0.090343982	3.66544E-05	-0.056501281	0.002262443
CYBRD1	-0.090225017	2.04296E-07	-0.229627772	0.002262443
DDX26B	-0.090091734	4.47241E-18	-0.020348457	0.019392372
PIK3CD	-0.0900271	1.1484E-26	-0.062585222	0.026826115
VIM	-0.089575516	2.64998E-12	-0.142571279	0.003878474
ARRB1	-0.089313674	4.66994E-15	-0.118446688	0.026826115

STON1	-0.08916762	1.8459E-16	-0.106620353	0.006140918
CCNI	-0.088879414	1.64878E-22	-0.070555918	0.000323206
GRK5	-0.088560914	4.70369E-25	-0.101144653	0.000646412
SMOC2	-0.088478367	0.000101914	-0.188643276	0.013574661
CD96	-0.087514474	5.48608E-16	-0.156523725	0.003878474
SCD5	-0.086890763	9.44451E-39	-0.070581981	0.006140918
TRPV4	-0.085361227	3.99134E-24	-0.055539606	0.026826115
HLA-DRA	-0.085186498	2.66487E-15	-0.1096818	0.006140918
C1QTNF7	-0.084921754	1.74023E-24	-0.108527658	0.000646412
CAV1	-0.08475315	2.52118E-05	-0.108813162	0.003878474
CD86	-0.084682543	8.38992E-18	-0.093116068	0.001292825
LIMCH1	-0.084124646	8.37554E-15	-0.093740683	0.00937298
RASL11B	-0.083785525	3.35758E-16	-0.293831584	0.001292825
TIMP2	-0.083530334	1.57018E-05	-0.110306965	0.002262443
NPDC1	-0.083389497	8.15711E-11	-0.080795591	0.00937298
ANTXR2	-0.082730036	1.14644E-16	-0.073315207	0.002262443
GALNT4	-0.082438652	1.14996E-13	-0.078549398	0.026826115
EML1	-0.082153934	1.51338E-18	-0.084028537	0.036522301
PRAM1	-0.082036753	7.29853E-18	-0.058700968	0.000323206
CAND2	-0.081947305	2.73563E-24	-0.03460849	0.006140918
CLEC7A	-0.081530132	1.21803E-35	-0.033603124	0.026826115
CLIP3	-0.081285659	2.42217E-11	-0.198686575	0.000646412
RAB3IL1	-0.081158976	1.03256E-16	-0.151958524	0.000646412
CSF1R	-0.081101282	3.16581E-19	-0.154221788	0.000323206
ISYNA1	-0.080445567	1.94313E-25	-0.117073522	0.001292825
TCEAL8	-0.080103798	0.004248671	-0.198220478	0.001292825
RGS10	-0.079571509	0.000466013	-0.15314579	0.000646412
IL15	-0.079090713	3.67041E-15	-0.037330098	0.019392372
GPR34	-0.078996238	0.001487533	-0.043428174	0.019392372
ASCL2	-0.078664212	7.55914E-16	-0.08655611	0.003878474
ITM2A	-0.078577421	0.001279079	-0.120466385	0.013574661
DNAH2	-0.078028055	3.189E-20	-0.09229221	0.001292825
TSPAN3	-0.077664739	1.70836E-05	-0.037242159	0.036522301
ZNF334	-0.077426581	5.25083E-16	-0.053992847	0.003878474
PHACTR2	-0.077290149	7.71473E-09	-0.061923011	0.00937298
HLA-DQB1	-0.077126372	1.51715E-06	-0.159669233	0.036522301
RGL1	-0.076879005	6.50518E-15	-0.130145425	0.001292825
SOD3	-0.076396676	5.97074E-10	-0.128473301	0.00937298
PARVG	-0.075970423	4.92565E-19	-0.068931921	0.036522301
CD19	-0.075933035	5.14702E-14	-0.154364208	0.006140918
GALC	-0.075582946	7.61243E-22	-0.109797586	0.003878474
CD37	-0.075393452	3.94546E-14	-0.111571592	0.002262443
CCDC8	-0.074342921	1.08002E-20	-0.059607358	0.019392372
HAND2	-0.074200203	1.29226E-17	-0.093210286	0.001292825
ZNF559	-0.073632513	3.27604E-05	-0.096196471	0.013574661
SPOCK2	-0.073216696	1.03281E-13	-0.171160165	0.003878474
CTNNAL1	-0.073176562	1.95694E-05	-0.086149306	0.002262443
COMMD6	-0.072789445	4.2295E-15	-0.047079609	0.003878474
PDZRN4	-0.072612902	1.06794E-18	-0.046438296	0.006140918
TMEM200A	-0.07155791	2.40479E-05	-0.114594109	0.048480931
VTCN1	-0.071429645	1.67316E-25	-0.30699979	0.006140918
TNFSF12-TN	-0.070954217	4.47026E-16	-0.03415354	0.036522301
MSRB3	-0.070703036	3.27604E-05	-0.104152092	0.00937298
HVCN1	-0.070056264	1.45778E-10	-0.044825714	0.013574661

UGT2A3	-0.069989289	6.40347E-07	-0.071969987	0.036522301
GPM6A	-0.069553345	2.09372E-16	-0.103984032	0.000323206
RPL26	-0.069509678	2.16797E-17	-0.116645711	0.00937298
HSD17B12	-0.06945426	2.60173E-05	-0.052609982	0.026826115
RAGE	-0.068491466	5.17465E-16	-0.124455872	0.001292825
CD84	-0.068406695	1.79708E-10	-0.050542192	0.013574661
BMP6	-0.0681536	2.09678E-19	-0.076803338	0.006140918
MAPK10	-0.067921661	1.00304E-15	-0.054841106	0.000646412
SPHK1	-0.067745894	1.11712E-09	-0.080138535	0.036522301
LY9	-0.067365867	1.98555E-17	-0.059342969	0.048480931
PIK3CG	-0.066801016	5.68239E-11	-0.110592949	0.001292825
PAPLN	-0.066528629	1.78558E-09	-0.176876666	0.001292825
CD6	-0.065965879	8.15108E-13	-0.145561708	0.026826115
VEGFB	-0.065868695	4.84943E-05	-0.060130739	0.000646412
PRDM1	-0.065237977	1.75488E-11	-0.084726898	0.002262443
VEPH1	-0.065219954	8.01654E-22	-0.059354887	0.026826115
LARP6	-0.065130081	8.26184E-09	-0.165384632	0.000646412
COPS4	-0.064435307	0.000143765	-0.064560782	0.000323206
LAYN	-0.064386119	5.55484E-08	-0.184378499	0.000646412
LRMP	-0.063111406	4.87174E-10	-0.125876449	0.000646412
ISLR	-0.062245543	9.32969E-16	-0.122699556	0.001292825
TMSB4X	-0.06213141	7.8352E-14	-0.033333988	0.013574661
CD209	-0.062109672	3.2874E-13	-0.08802151	0.013574661
GUCY1B3	-0.061913767	9.99261E-08	-0.077358893	0.002262443
SLC28A3	-0.061701059	3.77412E-12	-0.075615608	0.026826115
FXYD5	-0.061554528	5.476E-07	-0.094577979	0.001292825
INPP5D	-0.061537343	1.74032E-14	-0.073458836	0.019392372
EEF1G	-0.061317839	0.000256268	-0.087459392	0.036522301
CLEC11A	-0.061267518	8.58686E-18	-0.15529283	0.036522301
CLEC9A	-0.061181561	3.74893E-19	-0.045098774	0.002262443
RPS6KA5	-0.060983455	6.47936E-09	-0.042476961	0.002262443
PAM	-0.060918541	7.51676E-06	-0.119212041	0.00937298
CD79A	-0.060876068	6.62222E-13	-0.080763665	0.019392372
LMCD1	-0.060264083	1.72211E-05	-0.145181541	0.048480931
ENPP5	-0.059934008	3.02884E-16	-0.141936955	0.006140918
WDR86	-0.059905103	1.74032E-14	-0.116982984	0.006140918
SPRY1	-0.059880246	0.000262552	-0.107214563	0.003878474
FCGR2A	-0.059665499	0.000327091	-0.03456476	0.026826115
SH3GLB1	-0.059246032	3.32731E-05	-0.051138643	0.048480931
SAMD3	-0.059156397	2.57546E-12	-0.057353451	0.003878474
EMR2	-0.058886213	6.02958E-22	-0.064027859	0.003878474
P2RY8	-0.058671523	0.000179303	-0.132504444	0.00937298
ARHGAP4	-0.058317846	7.62788E-08	-0.127204681	0.000646412
S100A11	-0.057829826	0.014659601	-0.111276152	0.00937298
RCN3	-0.056423055	1.59977E-07	-0.090053496	0.006140918
RIC3	-0.05639702	5.96669E-28	-0.05521856	0.048480931
PCMT1	-0.055969614	1.98523E-07	-0.025418946	0.003878474
TGFB1I1	-0.055909675	6.3999E-08	-0.079673472	0.003878474
IFNGR1	-0.055710297	0.004775888	-0.040688839	0.013574661
CDH6	-0.055048594	5.51577E-11	-0.123185715	0.002262443
WNK2	-0.054854552	1.70831E-12	-0.056483849	0.006140918
FCHSD2	-0.054375829	3.25881E-17	-0.037667368	0.048480931
ZFP36L2	-0.054347	6.71048E-11	-0.109594033	0.019392372
COL12A1	-0.054089496	4.21606E-11	-0.042247951	0.001292825

SYTL1	-0.053722998	9.55636E-12	-0.063966487	0.013574661
ITGA4	-0.052654439	1.17979E-08	-0.047857953	0.00937298
PCDP1	-0.052529646	1.93471E-12	-0.105276272	0.001292825
EAF2	-0.052387482	9.32623E-07	-0.07740863	0.001292825
SLAMF1	-0.052324286	3.09651E-16	-0.104045312	0.006140918
TBXAS1	-0.052118241	4.7702E-19	-0.137707507	0.000646412
CMAH	-0.052092567	2.0668E-06	-0.058983703	0.026826115
VCL	-0.051922576	3.24439E-13	-0.076967087	0.006140918
ITM2C	-0.05183086	3.48733E-07	-0.284280276	0.001292825
PNRC2	-0.051688025	2.22606E-07	-0.032088112	0.019392372
ASNS	-0.05157589	0.000533898	-0.135999052	0.002262443
CRIP2	-0.05141924	1.50262E-07	-0.103448145	0.039711134
ADAMTS2	-0.051419112	2.96974E-12	-0.102648019	0.002262443
SFRS7	-0.051153928	2.17731E-20	-0.126838295	0.013574661
RPAIN	-0.050952465	3.44247E-13	-0.060925066	0.048480931
CSNK1A1	-0.050502415	1.06391E-16	-0.014980533	0.026826115
PTEN	-0.050275757	5.61152E-10	-0.053352871	0.006140918
SAMD9L	-0.050250726	0.001487533	-0.107209226	0.026826115
DHX15	-0.050100302	3.56121E-09	-0.032978366	0.019392372
SOX17	-0.050069514	9.61558E-12	-0.107554541	0.000646412
FUT3	-0.049991009	2.34985E-11	-0.049174244	0.048480931
FXYD6	-0.049914969	3.32744E-10	-0.111333782	0.003878474
PDGFC	-0.049889288	9.98123E-09	-0.103002325	0.002262443
GGT6	-0.049742138	4.54891E-17	-0.104865684	0.000323206
GABRP	-0.049710071	4.52605E-08	-0.158657452	0.001292825
ACSS1	-0.049626416	0.000138696	-0.141642159	0.019392372
SEC14L1	-0.0493911	2.46928E-10	-0.072306802	0.006140918
TMED3	-0.048917759	8.42163E-07	-0.183806332	0.019392372
NCF1	-0.048669641	1.18535E-12	-0.117732248	0.013574661
TMEM125	-0.04850947	3.07829E-09	-0.141500724	0.003878474
RERG	-0.048419852	3.42251E-07	-0.203428088	0.001292825
MCPH1	-0.048144609	4.87174E-10	-0.022092568	0.048480931
COL8A2	-0.047529842	1.10115E-10	-0.057528549	0.006140918
NAGK	-0.0473706	1.26902E-08	-0.061323383	0.030601606
CD68	-0.04732226	0.000142737	-0.033332477	0.036522301
CNN2	-0.047248742	2.36805E-07	-0.092765038	0.003878474
RPL36AL	-0.04694575	5.18471E-10	-0.024496973	0.00937298
PBX4	-0.046850121	1.35135E-10	-0.051478047	0.026826115
MYL9	-0.046658801	7.64451E-05	-0.144515979	0.001292825
PQLC3	-0.046456167	0.002744471	-0.078647193	0.00937298
TMEM43	-0.046405126	6.11602E-07	-0.072129797	0.006140918
YTHDC1	-0.046211917	1.13344E-18	-0.033184886	0.019392372
FBLN1	-0.045920859	1.19215E-08	-0.121400342	0.006140918
NPY5R	-0.045175894	2.57339E-11	-0.039202094	0.013574661
PLEKHB1	-0.045013643	1.73472E-15	-0.143827556	0.000646412
BCAS2	-0.044908703	0.000227702	-0.01847442	0.013574661
SEMA6A	-0.04450408	6.54656E-12	-0.132213517	0.013574661
RIN2	-0.044239566	7.06138E-06	-0.111549821	0.000646412
RPP25	-0.044111771	6.43292E-07	-0.127189354	0.000646412
FGFR3	-0.043544035	1.1729E-05	-0.082001954	0.00937298
FGD3	-0.043399078	1.69022E-09	-0.209086392	0.000323206
SVEP1	-0.043096744	2.77233E-14	-0.207789158	0.00937298
SLC44A2	-0.043052595	4.13165E-06	-0.155013133	0.000323206
ARHGAP25	-0.042980363	2.8345E-16	-0.041944023	0.019392372

ROR2	-0.042724536	2.58137E-05	-0.074588906	0.036522301
SPINT1	-0.042559834	2.91891E-14	-0.075097721	0.00937298
RPL21	-0.042431334	1.61499E-16	-0.082704849	0.003878474
MXRA7	-0.042417462	0.000108835	-0.057564247	0.013574661
RPL3	-0.042046017	2.26841E-12	-0.021545071	0.036522301
PRF1	-0.041977535	8.61946E-06	-0.068119459	0.026826115
TBRG1	-0.041971906	4.82108E-12	-0.020834136	0.026826115
PHLDB1	-0.041803492	2.49666E-11	-0.196227212	0.003878474
ITGB4	-0.041723172	3.5635E-10	-0.040031891	0.003878474
KIF17	-0.041718385	2.61681E-06	-0.06025173	0.036522301
CD1E	-0.04124349	3.56602E-18	-0.089838458	0.002262443
ANKS1A	-0.041200304	1.03356E-17	-0.118747002	0.003878474
RNF145	-0.041122962	4.73288E-09	-0.098397542	0.026826115
ARSJ	-0.041065043	4.93957E-09	-0.047289347	0.048480931
IL16	-0.040897133	2.91931E-16	-0.053818522	0.048480931
PRPS2	-0.040732936	0.003476189	-0.0429016	0.048480931
AMPH	-0.040596033	4.38574E-06	-0.061983876	0.000323206
SLC5A7	-0.040537708	6.79576E-09	-0.041402261	0.019392372
WWTR1	-0.040498869	0.000664972	-0.061426837	0.036522301
TSPAN32	-0.040430507	1.48308E-13	-0.092538145	0.000323206
MEI1	-0.040218699	2.40562E-20	-0.037397098	0.013574661
GDF2	-0.040101786	4.78023E-11	-0.073574858	0.026826115
PHACTR3	-0.040040838	2.13543E-12	-0.034170988	0.013574661
GPR132	-0.040026497	5.9038E-10	-0.053315921	0.003878474
TCF4	-0.039963367	0.010263484	-0.111095969	0.00937298
LSP1	-0.039520568	3.27748E-06	-0.016881478	0.019392372
GSTM5	-0.039332653	3.09872E-06	-0.092634864	0.036522301
HLA-E	-0.039295689	0.001806965	-0.052917792	0.036522301
OXCT2	-0.03911314	2.10107E-10	-0.192757403	0.001292825
MYO1G	-0.038859971	8.3907E-06	-0.101061941	0.000646412
GSTM3	-0.038795617	1.30643E-05	-0.216703065	0.003725294
ARRDC5	-0.038704842	1.03267E-08	-0.07068463	0.019392372
CLEC12A	-0.038311362	4.96937E-07	-0.080037142	0.002262443
CIRBP	-0.038304878	4.03603E-05	-0.055085144	0.00937298
NRP1	-0.038071725	1.78673E-07	-0.063828242	0.00937298
PLXDC2	-0.037816263	9.8685E-07	-0.072358901	0.00937298
CD33	-0.037544031	8.43763E-09	-0.128258835	0.000323206
RGL4	-0.037506421	1.35774E-05	-0.080767059	0.00937298
EMR1	-0.037426975	3.11547E-07	-0.0337685	0.019392372
COL6A2	-0.037412036	2.35683E-07	-0.081538721	0.026826115
RNASET2	-0.037065263	3.0072E-05	-0.061909473	0.000323206
SMAD9	-0.036974464	4.07906E-06	-0.052676609	0.026826115
NLGN2	-0.036328181	1.255E-09	-0.069533166	0.013574661
PHC1	-0.036283672	3.10138E-08	-0.070215153	0.013574661
ZNF135	-0.036128279	1.50608E-08	-0.085616802	0.036522301
PCNP	-0.035887638	1.6544E-05	-0.034352151	0.036522301
RPS3A	-0.035814276	1.32789E-10	-0.059779091	0.003878474
CLEC1A	-0.035761814	1.27564E-08	-0.076644403	0.00937298
DBC1	-0.035577607	9.15176E-12	-0.096718647	0.000646412
PLCB2	-0.035502783	1.38629E-09	-0.070885765	0.048480931
F2R	-0.035478053	4.30804E-05	-0.029479165	0.026826115
GEMIN4	-0.035400678	2.76892E-07	-0.101287497	0.000323206
TMC8	-0.035373922	8.29392E-08	-0.053074	0.013574661
MAP3K8	-0.035352699	2.02829E-05	-0.099425513	0.00937298

PRMT2	-0.03515709	0.000742392	-0.084838459	0.003878474
LEPREL2	-0.035118052	4.34444E-09	-0.067416255	0.013574661
GPM6B	-0.03509542	5.9361E-09	-0.03830499	0.013574661
ZNF586	-0.034652861	2.8247E-13	-0.068998331	0.002262443
YPEL4	-0.034510643	4.67873E-07	-0.045216243	0.003878474
PDGFD	-0.034368138	0.002342577	-0.21154407	0.003878474
PDE6B	-0.034367882	2.4095E-12	-0.039415121	0.036522301
CMTM3	-0.034324815	0.002335672	-0.06067248	0.013574661
SEMA4A	-0.03367943	1.72211E-05	-0.06641264	0.048480931
CCND3	-0.03346663	0.000310753	-0.112227902	0.003878474
SCRG1	-0.033232655	3.09038E-05	-0.16877613	0.000323206
CD69	-0.033121146	2.67382E-09	-0.176150615	0.006140918
RARB	-0.033089205	5.62966E-07	-0.05956743	0.036522301
RPL22	-0.033068315	2.25271E-07	-0.107846995	0.026763361
PLAC9	-0.033013827	0.002346037	-0.182534875	0.019392372
PRKACB	-0.032988878	7.96308E-05	-0.01518297	0.048480931
ENO2	-0.032815739	5.97242E-06	-0.096642873	0.003878474
LRRC49	-0.032717102	2.05542E-12	-0.112102136	0.000323206
JAM3	-0.032540269	0.000408992	-0.118395782	0.006140918
SIGLEC10	-0.032261953	5.55431E-09	-0.142221713	0.000646412
AFAP1L2	-0.032183166	6.79666E-07	-0.065961938	0.019392372
RAB8B	-0.032167778	8.63827E-05	-0.047168178	0.036522301
SLC15A3	-0.031934648	0.007138758	-0.049596453	0.019392372
OSBPL1A	-0.031743678	0.03393166	-0.101016988	0.000646412
ICAM2	-0.031704384	0.001936381	-0.078675405	0.001292825
L3MBTL3	-0.031426599	8.54311E-05	-0.013746421	0.048480931
LILRA5	-0.031311985	6.91554E-06	-0.117055768	0.000646412
GYPE	-0.031241161	3.16341E-05	-0.019417063	0.036522301
CTDSPL	-0.03122353	0.004430579	-0.095171833	0.00937298
ITGA8	-0.031206005	0.01250605	-0.044161413	0.036522301
RPS27A	-0.031032483	4.64321E-12	-0.031059213	0.036522301
INSL3	-0.030972215	0.000438831	-0.042915378	0.00937298
NTRK2	-0.030947672	5.03704E-12	-0.03397174	0.036522301
WDFY3	-0.03089704	0.000447723	-0.019342035	0.048480931
PROM2	-0.030804314	4.60328E-10	-0.130230266	0.006140918
MXRA8	-0.030707809	1.62834E-13	-0.089172756	0.002262443
GNG2	-0.030704431	2.27545E-05	-0.059284645	0.026826115
TINAGL1	-0.030351964	5.73963E-10	-0.052878208	0.019392372
SGCA	-0.030110121	4.76636E-07	-0.159845056	0.00186152
RAB11FIP5	-0.030023979	0.001942197	-0.074285988	0.048480931
RHOJ	-0.029822204	0.001187137	-0.05006456	0.026826115
RAB9A	-0.029732479	0.003711164	-0.038243233	0.039711134
GNB4	-0.029417255	1.88447E-06	-0.038118348	0.026826115
ADARB1	-0.029123071	2.21548E-07	-0.039621221	0.006140918
HMGCLL1	-0.028770564	6.02282E-06	-0.028176905	0.002262443
CRBN	-0.028658816	2.50535E-06	-0.048800226	0.000646412
ADARB2	-0.028567968	1.43179E-07	-0.043020937	0.048480931
INPP4B	-0.028470277	0.007936298	-0.06743985	0.001292825
PAFAH1B1	-0.028454704	3.57002E-07	-0.044166082	0.023348173
MUC1	-0.028445926	0.000519968	-0.078332402	0.003878474
RXFP1	-0.028262522	1.14981E-11	-0.049349699	0.002262443
CNOT7	-0.028241433	8.9958E-05	-0.03256984	0.048480931
ZNF630	-0.027983839	1.0324E-06	-0.07659651	0.000323206
LAMB1	-0.027735019	2.13575E-05	-0.093286515	0.00937298

RCN1	-0.0274934	0.001779905	-0.034028845	0.003878474
LGALS9	-0.027298125	7.96308E-05	-0.050060783	0.048480931
SLC31A2	-0.027032784	0.004492766	-0.090334632	0.000323206
AP1S2	-0.026822341	2.15304E-07	-0.105316666	0.002262443
BNC2	-0.026799442	1.31873E-07	-0.087064039	0.013574661
RPS12	-0.026468565	0.001948027	-0.031081592	0.013188555
VIPR1	-0.026134972	3.1256E-06	-0.233273348	0.006140918
PREX1	-0.026071679	0.037850828	-0.0482158	0.048480931
ART1	-0.02601738	0.000643672	-0.043729471	0.00937298
SPIB	-0.025951557	0.000498053	-0.059285234	0.00937298
SLFN13	-0.025928344	0.000593167	-0.070337344	0.000646412
GIMAP6	-0.025801094	6.1085E-05	-0.070280206	0.002262443
TRO	-0.025618299	1.07998E-06	-0.09967624	0.001292825
TGFB3	-0.025571077	0.000300289	-0.106384463	0.048480931
ERP29	-0.025449322	5.56003E-06	-0.089708441	0.000323206
NHS	-0.025325209	1.00483E-06	-0.017190627	0.048480931
HAPLN3	-0.025001554	0.008858734	-0.064926151	0.013574661
SLFN5	-0.024806457	0.04844768	-0.05532885	0.036522301
P2RY12	-0.02480044	9.89725E-05	-0.077968169	0.013574661
CTSW	-0.024548229	0.003011406	-0.099870157	0.048480931
FAM107A	-0.0244886	0.00042154	-0.161405301	0.013574661
RPS9	-0.024296132	0.000682481	-0.072167381	0.006140918
RRAS2	-0.024269075	0.002900231	-0.049992502	0.036522301
PLA2R1	-0.024232638	7.47616E-10	-0.039265455	0.036522301
NOTCH1	-0.02398051	6.73469E-07	-0.078110927	0.036522301
C2CD2	-0.0237172	0.021682539	-0.059365518	0.013574661
SFRP1	-0.023531197	0.00036098	-0.199031197	0.006140918
OR52E2	-0.023251006	0.004789133	-0.032229649	0.048480931
DDR1	-0.023186783	0.036002604	-0.049307696	0.026826115
ST3GAL5	-0.023179542	0.002477394	-0.043793974	0.036522301
APOBEC3H	-0.022978837	0.002846057	-0.07870154	0.003878474
IFNGR2	-0.022973957	0.025096194	-0.079315412	0.00937298
IQCK	-0.022951534	0.006209771	-0.079711044	0.019392372
SEMA6D	-0.022902566	0.004131191	-0.016981253	0.048480931
ANP32B	-0.022827919	6.93874E-05	-0.042745737	0.039711134
SHPK	-0.022757754	0.004492766	-0.072208904	0.036522301
PYCARD	-0.022721376	0.026879107	-0.075851856	0.036522301
EPHB6	-0.022705033	5.97212E-05	-0.136654969	0.003878474
MAP4K1	-0.022630794	0.006027792	-0.108616229	0.00937298
PCDH19	-0.022508394	1.28278E-05	-0.041730578	0.00937298
SEMA4D	-0.022375181	1.20292E-06	-0.182810257	0.002262443
FHOD1	-0.022337371	0.001721678	-0.079926554	0.007154102
SNCA	-0.022148085	5.32262E-09	-0.059104194	0.026826115
HSPB6	-0.022142946	0.001224678	-0.161099028	0.000646412
CACNA2D4	-0.022115428	1.40943E-09	-0.034634229	0.003878474
DGKA	-0.022007789	0.00038885	-0.046662878	0.003878474
APOBEC3F	-0.021887922	1.29324E-05	-0.069200519	0.001292825
MRC2	-0.021697691	0.007528063	-0.037642881	0.036522301
RARG	-0.021619786	0.000338425	-0.018130544	0.036522301
PCDH18	-0.02142064	0.009415627	-0.166968177	0.003878474
GFRA2	-0.021284158	0.00082679	-0.087959696	0.006140918
ASAM	-0.02120787	0.002528826	-0.055284888	0.000323206
RPL11	-0.021201908	3.92513E-06	-0.037698506	0.036522301
MRPL20	-0.020859152	0.000896911	-0.0740739	0.026826115

JAM2	-0.02081115	0.000102287	-0.094852595	0.00937298
KLHL6	-0.020705333	0.00284193	-0.064887436	0.036522301
C1QTNF5	-0.020653959	0.008812726	-0.150404109	0.00937298
GLI2	-0.020651768	1.07398E-08	-0.064244024	0.036522301
PIK3IP1	-0.020556218	0.024695227	-0.117655191	0.001292825
IL21R	-0.020545424	3.29163E-06	-0.015967536	0.048480931
COL13A1	-0.020477531	4.89579E-05	-0.048050155	0.00937298
NEO1	-0.020326261	0.002528826	-0.063333588	0.006140918
FCGBP	-0.020129447	0.00092002	-0.110124686	0.001292825
PTPN7	-0.019974641	3.07829E-09	-0.034922927	0.048480931
RBM4	-0.019961879	6.93874E-05	-0.024237986	0.036522301
TMEM18	-0.01994692	3.85392E-05	-0.044073166	0.019392372
RPL15	-0.019886326	0.026153227	-0.116886962	0.019392372
PFKM	-0.019621176	0.018108289	-0.061493067	0.003878474
IL2RA	-0.019515231	4.30804E-05	-0.031486242	0.002262443
PHACTR1	-0.019396503	4.11405E-06	-0.068753936	0.00937298
EFS	-0.019265984	0.000347757	-0.036208044	0.013574661
REM2	-0.019247746	0.005771667	-0.051717257	0.036522301
CCL24	-0.018910644	0.003208443	-0.059215115	0.000646412
PRDM8	-0.018771811	0.007428992	-0.061561669	0.036522301
NOD1	-0.018729542	0.005303174	-0.036147695	0.048480931
FABP3	-0.018552373	8.83159E-05	-0.05269986	0.048480931
CABP4	-0.018544301	0.039608223	-0.057915546	0.026826115
SGCE	-0.01853161	0.043239394	-0.227410204	0.048480931
RPL18A	-0.018531137	0.00341716	-0.054424113	0.036522301
SLC9A5	-0.018186973	0.039395433	-0.03226644	0.019392372
DDX43	-0.017920543	0.038180361	-0.058126822	0.003878474
EEF1A1	-0.017906358	3.16634E-06	-0.027344088	0.003878474
TMC6	-0.017542582	7.30086E-06	-0.100909132	0.002262443
ARHGEF6	-0.01753522	0.000532138	-0.163794239	0.001292825
GLTSCR2	-0.017475499	0.012951602	-0.055202399	0.007154102
GNGT2	-0.017275057	0.01088429	-0.050731538	0.019392372
CD4	-0.017148746	0.001571665	-0.081515753	0.026826115
HOPX	-0.016906472	0.009977876	-0.068847692	0.002262443
LRCH1	-0.016787972	0.002434072	-0.017718284	0.019392372
STXBP1	-0.016609275	0.001407568	-0.035652683	0.013574661
CNN1	-0.016494642	0.028127578	-0.081960996	0.026826115
PHF17	-0.016375451	0.006396681	-0.035258642	0.036522301
SPTLC1	-0.016296151	0.000530383	-0.03598602	0.002262443
PKNOX2	-0.016281676	0.000707258	-0.040536915	0.026826115
EMR3	-0.016171474	0.015434245	-0.030962584	0.048480931
DPYSL2	-0.016141486	0.022118148	-0.132849517	0.003878474
KCNMB1	-0.015842314	0.002267632	-0.121888813	0.000646412
OSBPL5	-0.015784693	0.001077412	-0.123021174	0.00937298
BICD2	-0.015629097	0.021682539	-0.035955254	0.006140918
ANKRD13A	-0.015398603	0.012165551	-0.033414795	0.00937298
ANKK1	-0.015376542	0.012983958	-0.033887014	0.00937298
ADC	-0.014619304	0.004296506	-0.037038202	0.036522301
IL12RB1	-0.014521659	0.000623008	-0.051233983	0.002262443
TEKT3	-0.014466776	0.014040452	-0.063113691	0.026826115
CD1A	-0.014219918	0.000846868	-0.045153533	0.036522301
SPNS3	-0.014084509	0.000791762	-0.053113739	0.006140918
ATRNL1	-0.013822422	3.73687E-05	-0.059955852	0.001292825
SLC1A5	-0.013790323	8.44895E-05	-0.094295448	0.026826115

JAG1	-0.013666635	0.009186847	-0.149008652	0.006140918
PABPC5	-0.013499558	0.024666786	-0.0213595	0.019392372
RPS15A	-0.013491706	0.002247577	-0.059963316	0.013574661
TTYH1	-0.013456555	9.09497E-06	-0.097823953	0.048480931
NBL1	-0.013305176	0.000984888	-0.129528696	0.003878474
CD8B	-0.013210912	0.001037516	-0.016834342	0.026826115
CCR2	-0.013062888	0.000849579	-0.044184041	0.00937298
OR13D1	-0.013059466	0.0415231	-0.036673864	0.019392372
TIE1	-0.012847875	0.002543699	-0.02644319	0.048480931
PTGS1	-0.01244909	0.005586059	-0.068081157	0.013574661
CCDC149	-0.012261841	0.028770339	-0.056040677	0.001292825
ZNF418	-0.011978589	0.015604847	-0.040718413	0.00937298
RPL32	-0.011913914	0.007215142	-0.027147328	0.013574661
ARHGEF2	-0.011845134	0.001552599	-0.098393474	0.00937298
ANKDD1A	-0.011550367	0.025269748	-0.071951838	0.003878474
MST1R	-0.011381793	0.038637441	-0.057806988	0.048480931
GBGT1	-0.011355923	0.005303174	-0.067837844	0.006140918
SMARCD3	-0.011287466	0.00858587	-0.069169285	0.002262443
ISCU	-0.011231804	0.004189557	-0.048820359	0.036522301
ARHGAP9	-0.01088486	0.004062125	-0.121487914	0.013574661
CD7	-0.010853514	0.023965282	-0.09367762	0.003878474
NKX3-1	-0.010473707	0.03639771	-0.070429197	0.019392372
USP51	-0.010169386	0.009926714	-0.054433524	0.013574661
SYDE1	-0.010111919	0.008041406	-0.113816373	0.001292825
RPS6KA2	-0.010099959	0.00471016	-0.04118561	0.048480931
NXN	-0.009940561	0.04134584	-0.106525705	0.019392372
RBM11	-0.009755696	0.034992404	-0.0337603	0.000323206
D4S234E	-0.009503274	0.030093878	-0.03548253	0.026826115
SCRN1	-0.009410887	0.022014973	-0.224117568	0.001292825
CASC1	-0.009292335	0.000572135	-0.097660274	0.000646412
SCN11A	-0.009205052	0.01021102	-0.023955501	0.036522301
MFGE8	-0.008678099	0.034043972	-0.193886463	0.001292825
CD28	-0.008453441	0.03884675	-0.037686057	0.036522301
ZNF256	-0.008422019	0.009306633	-0.068625598	0.026826115
RPL4	-0.007668383	0.008041406	-0.064513541	0.000646412
CPEB1	-0.007215558	5.79476E-05	-0.025527568	0.013574661
ZNF185	-0.007166486	0.022092321	-0.068454524	0.013574661
FHOD3	-0.006579913	0.000114523	-0.086394323	0.036522301
H2AFY2	-0.006540074	0.016887043	-0.25800949	0.007154102
Septin 4	-0.005538126	0.03153958	-0.088191102	0.002262443
EFCAB1	-0.005436313	0.002925549	-0.038207418	0.048480931
NFATC4	-0.004971182	0.006553351	-0.030367933	0.019392372
SGIP1	-0.004624784	0.000112052	-0.081681639	0.026826115
RTN1	-0.004557119	0.014623583	-0.042815766	0.036522301
DMKN	-0.002515484	3.79883E-09	-0.198127995	0.003878474
FBXL2	-0.001860995	0.003144511	-0.088141814	0.002262443
CA9	-0.001086806	7.31034E-10	-0.16113996	0.003878474
SULT1C4	-0.00017106	2.75685E-06	-0.081662918	0.048480931
PSMB1	0.00349854	0.00680394	0.019722374	0.013188555
APOC2	0.003676368	5.27341E-10	0.04732952	0.007118779
APOC1	0.005095477	0.000816265	0.035895213	0.005189033
APOA2	0.006635853	7.11606E-19	0.012026435	0.049322529
NDUFB4	0.007233353	0.028770339	0.018144622	0.019392372
NARFL	0.007794127	0.010939819	0.066413546	0.002262443

RARRES2	0.00807712	8.72704E-06	0.055578492	0.00937298
ZFYVE20	0.008090788	0.033485541	0.035366736	0.006140918
CTCFL	0.00873242	0.005962819	0.019206089	0.048480931
AAK1	0.008802348	0.022092321	0.02629528	0.026826115
STX1A	0.011299569	0.014587639	0.045138005	0.019392372
COX6B2	0.011338264	0.025444354	0.020311334	0.00937298
DPY19L2P2	0.011977816	0.025502785	0.029346073	0.013574661
TOMM40L	0.012100706	0.002294627	0.083570452	0.003878474
PCDHGB3	0.012242462	0.02609351	0.034692735	0.036522301
HNF4G	0.012534082	0.031750896	0.047652181	0.036522301
MORC1	0.012609944	0.049674009	0.046305126	0.019392372
RAD52	0.012838558	0.000860503	0.021146567	0.026826115
FBXO10	0.01327801	0.010746559	0.030950389	0.00937298
KIAA1199	0.013635074	0.027685043	0.199334207	0.048480931
CFHR4	0.014213807	0.028900398	0.198947566	0.026826115
POT1	0.014307491	0.041790196	0.021238538	0.026826115
CNOT3	0.014551854	0.008744113	0.052200262	0.026826115
MAPRE3	0.014813247	0.001685542	0.069167223	0.00937298
PIWIL3	0.014849131	0.005946674	0.023036237	0.036522301
RGAG1	0.015401488	0.010502549	0.064631544	0.013574661
GFM1	0.015732021	0.028576196	0.051874309	0.026826115
LRP1	0.015758173	0.013612455	0.062713849	0.048480931
OR10Q1	0.015766681	0.007331093	0.035930802	0.013574661
SKIV2L	0.016043582	0.000289161	0.066202779	0.006140918
PPM1L	0.016201369	0.028770339	0.027718496	0.048480931
TMEM180	0.016538991	0.003055194	0.042006189	0.036522301
TAF8	0.016592991	0.00359704	0.043358647	0.003878474
GBF1	0.016822968	0.007648511	0.046538183	0.048480931
ZNF407	0.016943905	0.002752474	0.015767153	0.002262443
PI15	0.017120812	0.004762677	0.052891562	0.003878474
GDI1	0.017561381	0.002342577	0.048213664	0.019392372
ALDH1A1	0.017827326	7.0909E-06	0.03475527	0.019392372
IQGAP3	0.017959094	0.000934742	0.052416138	0.013574661
SLC2A10	0.018233219	0.011136158	0.063658028	0.019392372
RAB11FIP3	0.018296743	0.00420132	0.045385576	0.048480931
HLCS	0.018733262	0.02137957	0.039249931	0.048480931
HIST1H2BF	0.01878729	0.00678576	0.042387272	0.026826115
IRF3	0.018789348	0.03639771	0.044425697	0.019392372
DHRS3	0.018906556	0.025737672	0.037783948	0.006140918
GLI4	0.019705242	0.002201412	0.061238084	0.00937298
FAM73B	0.019709984	0.043608257	0.066064808	0.019392372
RXRA	0.019732282	0.02975818	0.072314219	0.026826115
PTPRA	0.019929455	0.024895004	0.024392559	0.019392372
MLL4	0.020238674	0.03146941	0.073998894	0.026826115
HELZ	0.020910566	0.028867828	0.085035168	0.019392372
ULK3	0.02108992	0.002363408	0.070882248	0.026826115
AKT2	0.021204818	0.000555445	0.047765801	0.006140918
KTN1	0.02147492	1.35225E-05	0.032383313	0.048480931
CLCN2	0.02162301	0.000519968	0.027519887	0.019392372
ZNHIT1	0.021716512	0.00014532	0.061881919	0.00937298
TUFT1	0.021918734	3.5414E-06	0.073489474	0.003725294
MRRF	0.022173437	0.010145773	0.023837618	0.003878474
ZSWIM3	0.022792714	6.6966E-05	0.021045552	0.00937298
CCDC56	0.023070383	0.000167023	0.025524737	0.026826115

DNM2	0.023452377	0.002111679	0.037798877	0.048480931
ZSCAN1	0.023456121	0.000288168	0.046083969	0.036522301
MTMR10	0.023622381	9.68196E-05	0.061647585	0.001292825
SEC61A2	0.023733047	0.000340736	0.058139459	0.019392372
FH	0.0239613	0.000268053	0.068927983	0.019392372
SCYL3	0.023982883	0.018792211	0.027129799	0.026826115
SAMD4B	0.024018749	4.47609E-05	0.096131938	0.00937298
NFYA	0.02452225	0.000754459	0.031774484	0.026826115
TAF15	0.024596805	4.40446E-06	0.045980478	0.048480931
SUPT6H	0.024903873	0.000477002	0.047380915	0.001292825
F8A1	0.025105835	0.00306402	0.047899791	0.039711134
SIGLEC15	0.025175376	0.000407619	0.038543626	0.019392372
GAA	0.025223395	8.32492E-05	0.091411343	0.000323206
EXOC5	0.025703968	5.37293E-05	0.024212917	0.036522301
FTL	0.025894735	2.05559E-30	0.00939975	0.02031785
HSPA9	0.025949329	0.000176781	0.053876102	0.003878474
KLHL34	0.026054499	0.003861171	0.03356565	0.048480931
HIPK2	0.026077463	0.002462874	0.126364149	0.013574661
CGN	0.02673147	0.003711164	0.149926094	0.003878474
KIAA0895	0.026875708	0.000509744	0.101738392	0.036522301
ZNF146	0.027095556	2.9548E-06	0.030931494	0.013574661
YTHDF2	0.027247927	3.36627E-05	0.030038866	0.006140918
ANXA7	0.027441163	6.57045E-10	0.029086689	0.036522301
RAB43	0.02749404	0.000259841	0.044443901	0.026826115
SLC12A7	0.027704712	0.00035371	0.022832287	0.048480931
CHERP	0.02770616	2.23089E-05	0.08301814	0.000323206
RREB1	0.027800109	5.70407E-09	0.038200783	0.006140918
CYCS	0.027843254	0.041169222	0.069218007	0.036522301
DIAPH1	0.027966113	2.56116E-05	0.08607111	0.003878474
AGBL3	0.028061996	0.018994962	0.044690804	0.026826115
DNAJC3	0.028125984	0.010773984	0.080681747	0.00937298
CLTCL1	0.028163903	0.02787396	0.099973272	0.00937298
TTC31	0.028477031	2.27596E-06	0.062655065	0.036522301
TSC22D4	0.029037779	0.000983333	0.045693256	0.048480931
MRPS25	0.029233912	0.006713474	0.081829286	0.036522301
ZDHHC18	0.029500511	0.009186847	0.053842844	0.048480931
CYP11A1	0.029631703	0.001706105	0.166539582	0.019392372
LZTR1	0.02972496	7.80332E-06	0.057118027	0.019392372
SAMD10	0.029921429	0.000382345	0.041485438	0.013574661
DCTN4	0.030464799	0.003245505	0.043125518	0.036522301
PPIL2	0.030663968	0.000225331	0.022176157	0.002262443
TMEM143	0.030678272	3.24935E-06	0.075257594	0.026826115
WBSCR27	0.030837373	5.32637E-07	0.106585831	0.019392372
IL32	0.030884598	0.024809219	0.133267449	0.003878474
HEXB	0.030900912	4.91329E-10	0.040114255	0.003878474
RSAD1	0.031005852	1.92795E-09	0.117322571	0.003878474
FAM40B	0.031038531	0.000390164	0.056697701	0.00937298
PSME4	0.031091677	4.70445E-06	0.072108645	0.002262443
UPF2	0.03122355	0.000250994	0.050459902	0.00937298
TRIP11	0.031605834	0.008277043	0.044815443	0.036522301
ZNF513	0.031718237	0.044447926	0.087843898	0.000646412
SPTAN1	0.031806943	2.33927E-05	0.040413444	0.048480931
PANX2	0.032089024	9.87578E-06	0.22786885	0.000646412
TSC2	0.032172515	2.40479E-05	0.061564027	0.006140918

WDR26	0.032597563	5.97242E-06	0.099627614	0.000323206
UQCC	0.032611964	4.99243E-07	0.034479505	0.026826115
DHX34	0.033297443	1.01899E-07	0.043285754	0.003878474
VDAC1	0.033361428	1.32016E-10	0.035795296	0.00937298
MYO5B	0.033813228	2.41348E-07	0.065807921	0.036522301
ARFGAP1	0.034008901	1.58924E-05	0.055683199	0.002262443
AUP1	0.034721653	1.88447E-06	0.037886633	0.002262443
OGFR	0.03476262	3.85392E-05	0.05870551	0.019392372
ARHGEF11	0.034791438	8.34929E-09	0.046650851	0.000323206
KIAA0556	0.034933709	3.07836E-05	0.062151711	0.019392372
RBM33	0.035117856	8.35531E-08	0.065464579	0.048480931
GGA3	0.035179553	1.25631E-07	0.029012207	0.013574661
MFSD9	0.03540073	0.00019723	0.089995399	0.006140918
SLC38A10	0.035635588	9.89725E-05	0.057650342	0.003878474
UBE4A	0.035646049	8.60192E-11	0.060541261	0.002262443
YWHAZ	0.035688449	0.000258049	0.037246716	0.036522301
FAF1	0.035766103	1.87716E-15	0.032392747	0.006140918
HUWE1	0.035786685	4.72348E-11	0.071416608	0.000323206
VAPB	0.035993377	1.579E-06	0.094543812	0.019392372
CNOT4	0.036298909	1.67264E-06	0.04225369	0.002262443
GON4L	0.036498798	7.54809E-06	0.03123203	0.036522301
MRPS5	0.036661323	5.69491E-07	0.047459242	0.026826115
CLEC16A	0.036687378	6.00449E-10	0.053634874	0.026826115
POLM	0.036761428	2.71771E-09	0.066248307	0.048480931
RRM2B	0.037060303	5.57798E-07	0.043376934	0.019392372
MXD3	0.037121413	3.59531E-05	0.072616758	0.000646412
NDST1	0.037444597	3.47227E-05	0.076668586	0.026826115
IGF2R	0.037536072	3.18963E-07	0.035571745	0.013574661
SUPT7L	0.037658762	2.66272E-06	0.017719872	0.048480931
TMEM64	0.037696975	0.001515105	0.062359697	0.006140918
CCDC109A	0.037750156	9.91644E-06	0.069309877	0.001292825
NF1	0.038033822	5.7504E-11	0.038761794	0.02031785
KLHL8	0.038228645	0.016887043	0.059291027	0.00937298
PYCRL	0.038443117	4.27514E-09	0.036846038	0.036522301
GOLGA3	0.038697178	1.86803E-12	0.061310086	0.019392372
EIF5B	0.038745611	1.03548E-11	0.027429636	0.036522301
UQCRB	0.038775989	0.005420668	0.067785681	0.019392372
SDCCAG3	0.039063707	5.49014E-06	0.042400498	0.048480931
UBE2V2	0.039104447	0.034231887	0.031798825	0.006140918
DENND2C	0.03927173	0.000398132	0.046403417	0.026826115
HOXA6	0.039457388	1.66774E-05	0.038752003	0.026826115
TRPC4AP	0.039512441	5.87283E-06	0.046235349	0.026826115
POP7	0.039714162	8.45586E-10	0.084446602	0.026826115
ADCK4	0.03990944	0.002156117	0.089084477	0.003878474
VPS39	0.040193702	3.08569E-08	0.040391183	0.013574661
OTUD6B	0.04033348	0.000121806	0.049714084	0.003878474
USP19	0.040440344	8.62726E-17	0.060145257	0.006140918
MRPL22	0.04051811	1.78639E-18	0.018303057	0.019392372
NFIC	0.040788439	3.55384E-05	0.044406843	0.048480931
TYW1	0.040828271	0.000400821	0.0550842	0.036522301
MYH14	0.040906968	1.18153E-06	0.085470946	0.013574661
MUS81	0.041067423	7.44736E-07	0.04172072	0.002262443
BLVRB	0.041220715	3.64609E-08	0.058737339	0.036522301
HERC2	0.04143127	3.86557E-07	0.037168635	0.00937298

PDIA4	0.041850371	1.08045E-07	0.081028533	0.019392372
GGNBP2	0.042045714	2.61356E-17	0.039150397	0.019392372
DNA2	0.042120485	1.98835E-05	0.058037548	0.000323206
NGEF	0.042128992	9.75471E-06	0.06343918	0.036522301
CHD8	0.042246526	3.40565E-05	0.057958616	0.019392372
HDLBP	0.042564922	1.10131E-11	0.015762293	0.048480931
RAF1	0.042570591	2.04448E-05	0.041821638	0.013574661
ULK1	0.042623247	0.001505862	0.039139687	0.00937298
TSC1	0.042637402	4.78233E-08	0.033042352	0.048480931
FOXD2	0.04274729	7.83747E-08	0.165396817	0.000646412
APBB3	0.043210772	0.00058929	0.097410384	0.048480931
PDDC1	0.043241029	1.95541E-17	0.064253251	0.000323206
ZNF622	0.043409163	6.2547E-06	0.03380733	0.036522301
POLR3A	0.043552904	1.40081E-12	0.042816447	0.001292825
SEC24C	0.043626175	5.13247E-12	0.045084358	0.003878474
RG9MTD2	0.043631436	0.001693224	0.072571316	0.006140918
DAG1	0.043825877	3.79794E-12	0.034595624	0.002262443
MTERFD3	0.044236122	1.9844E-05	0.026899374	0.00937298
YTHDF1	0.044777221	8.89322E-09	0.040479718	0.013574661
DGCR14	0.045001507	2.48353E-10	0.034880222	0.000646412
UCN	0.045430141	9.57597E-05	0.107035912	0.006140918
DHX30	0.045497274	5.21891E-06	0.024636388	0.001292825
ATG9A	0.045510963	7.50953E-11	0.048063323	0.00937298
WDR24	0.045647253	5.07426E-11	0.064484003	0.000323206
PLEKHM3	0.045871026	0.000236599	0.06203197	0.013574661
ACBD4	0.046143049	0.002875112	0.053959326	0.036522301
ATP6V0A1	0.046188292	2.50687E-07	0.095010194	0.026763361
CCDC117	0.046229657	5.51577E-11	0.043573598	0.003878474
NR1D2	0.046526595	0.015172118	0.038573096	0.026826115
GTF3C1	0.046648205	3.31186E-07	0.05932383	0.013574661
PTCD3	0.046689102	1.39343E-08	0.064792225	0.000646412
AGPAT1	0.046742499	2.64998E-12	0.041996302	0.006140918
SF3B2	0.047044148	7.94757E-18	0.039827481	0.003878474
KIF21A	0.047077077	6.10524E-08	0.106566727	0.000323206
STAG3L1	0.047163584	1.53937E-09	0.055229849	0.00937298
DTNA	0.047213255	1.17019E-12	0.140435709	0.048480931
TTC30A	0.047333379	2.99318E-07	0.049749267	0.00937298
ESRRA	0.047500342	3.78036E-05	0.114592037	0.003878474
LRPPRC	0.047662106	1.34791E-12	0.047224645	0.048480931
NSUN3	0.04766887	2.10212E-05	0.048556213	0.000323206
TOMM70A	0.047796376	5.19675E-11	0.041409288	0.015270486
SF4	0.047898633	9.71682E-18	0.031631336	0.019392372
RTN4IP1	0.048095364	2.51874E-09	0.055483759	0.036522301
SURF6	0.048101058	5.66656E-08	0.052205852	0.036522301
OPA1	0.048237166	3.05377E-10	0.020877979	0.019392372
ATP2B2	0.048652836	1.92642E-06	0.126916371	0.000323206
KCTD3	0.049132671	7.1276E-15	0.042902703	0.019392372
BAP1	0.049349043	3.70075E-13	0.088103229	0.000646412
WNK1	0.049429655	4.14736E-12	0.068071536	0.001292825
CDK5RAP3	0.049576488	2.63597E-08	0.075438706	0.002262443
TTC7A	0.049612272	0.003556338	0.034446567	0.048480931
GBA2	0.050257233	9.39994E-06	0.041884415	0.026826115
ALG6	0.050631547	9.02752E-14	0.045064836	0.026826115
NRD1	0.050948069	4.72417E-17	0.049921934	0.006140918

HMGA1	0.050972325	1.25241E-06	0.01933933	0.013574661
SDAD1	0.051223882	7.73875E-06	0.02745364	0.013574661
DIO2	0.051302284	0.010746563	0.119908828	0.00937298
ERO1LB	0.051647783	0.000486613	0.066564204	0.002262443
SHOX2	0.0517769	2.78204E-07	0.024101073	0.003878474
SLC23A1	0.052014437	0.000369639	0.07518898	0.00937298
RAB2A	0.052143195	4.11761E-09	0.065827983	0.013574661
TFG	0.052369952	5.11919E-15	0.046695999	0.00937298
RBM28	0.052473663	3.29569E-08	0.08836553	0.002262443
SATB2	0.052497017	5.6437E-09	0.155537622	0.036522301
ZNF672	0.052575692	2.01662E-15	0.077141153	0.002262443
UBFD1	0.053150974	3.63985E-11	0.065531094	0.019392372
IKBKG	0.053196509	7.39557E-13	0.091467789	0.013574661
SLC35E1	0.053524257	6.57004E-19	0.031674542	0.026826115
MET	0.053689268	1.77248E-15	0.078750521	0.036522301
OCRL	0.053751552	2.82127E-15	0.086190181	0.00937298
ZHX3	0.053969998	1.39113E-05	0.072860664	0.036522301
MLH3	0.054339966	6.17604E-10	0.060183818	0.000646412
DUSP8	0.054340236	0.001072348	0.048857762	0.036522301
NELF	0.054982658	2.13594E-06	0.065762311	0.048480931
MTF1	0.055049377	3.60362E-07	0.072888225	0.019392372
PTPLB	0.055184338	4.78852E-07	0.048312903	0.003878474
GSS	0.055443358	3.48574E-05	0.056182856	0.006140918
ENY2	0.0555431	1.16078E-10	0.050011244	0.026763361
CDC42BPB	0.055969401	8.70226E-05	0.093863877	0.000323206
LPIN3	0.055974714	0.00408503	0.04023749	0.026826115
FBXL20	0.055982093	5.15546E-10	0.054145354	0.026826115
MYRIP	0.056317818	0.000282281	0.046764581	0.019392372
UCK1	0.05642272	8.112E-05	0.059592922	0.026826115
IL6R	0.05643906	6.075E-08	0.082372866	0.019392372
ANKZF1	0.05688062	1.66152E-08	0.034675843	0.048480931
HSD3B7	0.056938107	9.89537E-08	0.142599758	0.006140918
GTF3C3	0.057213729	3.89599E-21	0.058042335	0.003878474
DHX35	0.0573522	1.87247E-24	0.055881611	0.013574661
GCN1L1	0.057535134	3.83045E-11	0.061153287	0.039711134
BAT2	0.057658849	1.4255E-11	0.040643958	0.013574661
TRAF2	0.057755906	2.93902E-14	0.058816099	0.019392372
STIM1	0.05777951	1.93177E-15	0.096012166	0.000323206
RHBDD3	0.058236701	2.71773E-08	0.105854557	0.000646412
HGS	0.058239181	2.98858E-12	0.04147041	0.013574661
DPP4	0.058320146	9.43874E-06	0.113006486	0.048480931
SLC45A2	0.05841802	0.000231707	0.065697215	0.026826115
ABHD8	0.05854429	1.214E-09	0.07183134	0.001292825
TULP4	0.058610866	3.76433E-17	0.04180598	0.026826115
PA2G4	0.058625312	5.10674E-23	0.03585997	0.019392372
SGTA	0.058803497	1.24523E-20	0.053423459	0.006140918
UPF1	0.058842455	7.032E-14	0.083666049	0.036522301
P4HB	0.059252741	9.39979E-14	0.062714161	0.013188555
CHD4	0.059531279	1.08316E-12	0.052274907	0.00937298
GPKOW	0.059650608	1.38414E-07	0.041188617	0.048480931
PSMD1	0.059776751	2.56016E-26	0.055632558	0.036522301
ATP2A2	0.059921413	1.54202E-17	0.073537874	0.003878474
LONP1	0.059945595	1.12948E-12	0.053757627	0.002262443
FBLN7	0.060203489	4.76257E-10	0.090103547	0.003878474

TAPBP	0.060588502	5.11919E-15	0.031552829	0.048480931
VPS26B	0.061236936	3.71005E-14	0.060986158	0.011361673
AKAP8L	0.061270513	7.00269E-06	0.057205679	0.036522301
GNPTG	0.0614749	3.02885E-16	0.045874519	0.00937298
TMUB1	0.061652209	9.90725E-11	0.074529883	0.026826115
LOC727726	0.061828493	3.18904E-16	0.025399327	0.036522301
SAPS1	0.061866474	1.04451E-10	0.060311643	0.000646412
ADCK2	0.062091803	3.25799E-09	0.061447167	0.048480931
YTHDF3	0.062095808	3.53656E-14	0.037952615	0.019392372
TUBGCP4	0.062318471	9.59548E-06	0.049780288	0.006140918
AP3D1	0.062629037	1.39552E-17	0.036576582	0.003878474
PSMC3	0.062851502	7.40068E-25	0.037135749	0.013574661
TTC1	0.063045231	2.92458E-26	0.04160239	0.036522301
PDPK1	0.063119895	2.44026E-17	0.019363128	0.026826115
CLPTM1L	0.063356532	4.84216E-23	0.088021745	0.026826115
COX6C	0.063614684	5.3261E-31	0.046648949	0.008328186
NARF	0.063825922	9.39659E-23	0.063660072	0.036522301
TOM1	0.064032807	7.05009E-07	0.049475356	0.013574661
SLC25A30	0.06430848	3.54274E-11	0.039917651	0.048480931
B3GAT3	0.064455244	3.26251E-08	0.052404096	0.019392372
PPFIA1	0.064550714	5.60757E-16	0.024772635	0.019392372
YIPF6	0.064585958	2.76283E-14	0.034262134	0.036522301
CRYGS	0.064726541	0.000286193	0.079061891	0.036522301
PSMC2	0.064747718	1.09718E-13	0.064584313	0.013574661
CYP4F11	0.064793958	1.54672E-07	0.10305185	0.013574661
SDHC	0.064956326	6.20498E-13	0.053697627	0.048480931
CD99L2	0.064966385	1.30223E-17	0.086306033	0.036522301
DYSF	0.065115081	1.38414E-07	0.082084562	0.003878474
WBSCR16	0.065187182	3.74278E-23	0.034565965	0.048480931
VARS2	0.065459482	1.48115E-09	0.057494346	0.039711134
XPO1	0.0654795	3.29614E-17	0.033366244	0.048480931
TRIM41	0.065522713	5.56678E-16	0.065913562	0.002262443
TJP1	0.065597622	4.30805E-05	0.077134825	0.006140918
CRELD1	0.06584958	5.44402E-06	0.02916407	0.00937298
SLC6A1	0.065881885	0.004381392	0.118382492	0.048480931
LRRC2	0.065966392	0.000219887	0.051588783	0.003878474
PARP12	0.06611213	1.2957E-08	0.109652503	0.00937298
KIF3B	0.066516419	4.42697E-30	0.046636479	0.006140918
TACC2	0.066834523	1.29124E-14	0.070858963	0.006140918
NUDCD3	0.067064703	2.29239E-23	0.054424366	0.048480931
ATP5SL	0.067599379	3.90271E-19	0.025015339	0.036522301
TOR1AIP2	0.067706722	6.43758E-11	0.065471025	0.013574661
EFTUD2	0.067862001	1.12898E-23	0.033513032	0.026826115
VPS13A	0.067929531	3.0124E-09	0.046442262	0.001292825
SCLY	0.068347147	1.42418E-10	0.069967374	0.036522301
DHX29	0.068434849	1.4153E-13	0.058585106	0.000323206
KIAA1543	0.068573511	2.18405E-07	0.074971296	0.003878474
OLA1	0.068626898	3.67154E-21	0.01642353	0.036522301
NKIRAS2	0.069091077	4.11623E-11	0.027024469	0.00937298
EDEM3	0.069565308	1.59145E-15	0.047355052	0.001292825
PARP10	0.06965192	1.19526E-08	0.068541357	0.002262443
TBCD	0.069724919	3.79818E-21	0.039040815	0.036522301
UTP6	0.069827018	0.008518839	0.04848353	0.003878474
MCFD2	0.069907222	7.8182E-08	0.045932509	0.013574661

ZIC2	0.06991516	1.75426E-19	0.183148651	0.000323206
EGLN1	0.070256425	4.75179E-11	0.083050564	0.019392372
GTF2H4	0.070404584	1.01078E-09	0.055214743	0.036522301
RALGPS2	0.070446443	0.000125383	0.069879551	0.006140918
TALDO1	0.070717986	9.84867E-25	0.038659889	0.003878474
SLC38A3	0.070723601	0.000325977	0.13247479	0.003878474
ITLN2	0.070775447	0.000110031	0.127373546	0.003878474
CDC25B	0.070930775	1.52251E-09	0.050465114	0.003878474
DDB1	0.071360226	2.74049E-23	0.024604087	0.013574661
POLR3G	0.071413764	2.91942E-21	0.023623223	0.048480931
ADRM1	0.071540326	5.12703E-19	0.047674663	0.019392372
ZNF282	0.071837047	4.07999E-30	0.074000808	0.001292825
DTX2	0.072102396	2.29664E-08	0.033380757	0.019392372
MGAT4B	0.072191656	2.19549E-20	0.068488209	0.006140918
UBE3B	0.072420843	3.06152E-29	0.04597009	0.019392372
PCGF1	0.072573931	1.54201E-17	0.062177798	0.00937298
BRD3	0.073031298	1.4488E-13	0.05724094	0.00937298
ZXDB	0.073165268	2.59415E-06	0.077587383	0.004402322
MAST2	0.073379535	2.78774E-08	0.028933056	0.003878474
ITPK1	0.073685509	2.87384E-19	0.138993639	0.001292825
KIAA1598	0.074553639	9.84919E-11	0.103643753	0.002262443
BRF2	0.074560989	5.99479E-11	0.083901253	0.00937298
ATOX1	0.075077627	1.21659E-19	0.063615722	0.002262443
NSFL1C	0.075080437	1.79884E-30	0.054041536	0.019392372
DPP9	0.075416963	4.55018E-07	0.092447464	0.026826115
MTDH	0.075437787	6.96685E-18	0.07028045	0.026826115
HDGF	0.07592902	7.96671E-11	0.041373461	0.019392372
EIF3B	0.07613244	3.36034E-30	0.03629776	0.036522301
NRBP2	0.076404662	9.26561E-12	0.119633673	0.039711134
TXN	0.076800092	5.123E-27	0.060747243	0.00937298
IARS2	0.07708615	6.67583E-19	0.044629846	0.019392372
ZKSCAN1	0.077315482	3.13889E-05	0.099322005	0.036522301
DPP3	0.077526338	1.8231E-08	0.062085571	0.036522301
TBC1D5	0.077532529	3.94758E-27	0.039344181	0.00937298
COPG	0.078339288	3.24327E-19	0.028581308	0.026826115
PSMD11	0.078833607	3.60113E-19	0.050401401	0.013574661
DDX56	0.079106727	3.82528E-20	0.062565665	0.013574661
EIF2B4	0.079150003	8.74045E-16	0.026200262	0.026826115
ASB8	0.079518729	3.88164E-10	0.052380592	0.000323206
MEPCE	0.079673268	2.99484E-26	0.075185347	0.026826115
QSOX2	0.080574287	1.41679E-11	0.072391498	0.003878474
STIL	0.080940049	1.01723E-16	0.098702847	0.00937298
ELAVL1	0.081441556	1.66805E-20	0.080060475	0.003878474
AKAP1	0.081483035	2.37579E-23	0.041253971	0.013574661
ZKSCAN5	0.08180236	2.27054E-36	0.025491794	0.048480931
NAPRT1	0.081861617	4.29247E-11	0.087970804	0.000646412
TRIO	0.081949888	5.66262E-17	0.073763801	0.00937298
UFD1L	0.082161255	8.33344E-22	0.052176663	0.026826115
SEMA6C	0.08239169	4.00856E-09	0.068052884	0.026826115
TOMM34	0.082473159	8.78836E-18	0.036351623	0.048480931
SLC38A9	0.082509356	2.71798E-12	0.050849493	0.036522301
KIAA1715	0.082806071	1.55641E-09	0.040121609	0.036522301
NUCKS1	0.08284236	3.27391E-28	0.083009519	0.013574661
ATXN7L3	0.083181865	2.07987E-19	0.060307873	0.019392372

TRIM26	0.083223509	1.23796E-10	0.034665833	0.019392372
ADAR	0.083230912	2.43447E-28	0.023241667	0.036522301
BAZ1B	0.083671472	1.01339E-23	0.043208361	0.013574661
PRAP1	0.083720583	1.30612E-13	0.114635634	0.002262443
PTPN23	0.083757722	3.99391E-12	0.051907224	0.013574661
NR2F6	0.083821651	3.62425E-17	0.08748215	0.003878474
SPC24	0.084288517	3.31472E-22	0.094985748	0.008368042
TOMM40	0.084524706	5.34087E-13	0.041564044	0.006140918
GSK3B	0.084643495	2.6282E-36	0.044972905	0.006140918
ZFYVE19	0.084686052	1.48794E-11	0.10914488	0.000323206
CRTC2	0.084925725	1.1339E-10	0.059529493	0.019392372
ADCY6	0.085149012	3.73163E-20	0.071607182	0.001292825
ATP5J2	0.086394915	9.8726E-41	0.057468586	0.036522301
NPLOC4	0.086553187	6.60134E-26	0.144006354	0.000646412
IQSEC1	0.08670058	2.83607E-05	0.080518552	0.000646412
DBNDD1	0.087278258	2.73216E-18	0.15392992	0.026826115
BCL2L12	0.087625652	6.50305E-25	0.054008225	0.013574661
VARS	0.087839397	2.27736E-25	0.040511121	0.000323206
PTPRF	0.087876845	1.27998E-27	0.112349539	0.00937298
LASS2	0.088348562	8.75454E-08	0.09177559	0.000323206
UCHL5	0.088441792	9.95294E-24	0.034652492	0.048480931
MSH6	0.088654409	6.07503E-12	0.04241182	0.026826115
PRKCA	0.088981441	2.35324E-24	0.081957156	0.00937298
COG4	0.089070711	1.52669E-21	0.057369626	0.026826115
PCBD2	0.089222333	7.89119E-13	0.075721436	0.000646412
NUBP2	0.089571093	5.58335E-24	0.034770789	0.036522301
DIS3L2	0.089762473	5.41411E-26	0.062384142	0.019392372
ZSCAN29	0.090040353	5.67958E-23	0.064736053	0.00937298
PTDSS1	0.091079427	3.55525E-08	0.05211771	0.026826115
PUS1	0.091754133	1.43177E-19	0.033696308	0.013574661
SLC35A4	0.091824832	7.24218E-18	0.055778549	0.019392372
HYOU1	0.091928216	1.40048E-25	0.117839332	0.006140918
THOC2	0.092594437	7.21061E-27	0.035782395	0.036522301
TMEM189	0.092822007	5.14751E-20	0.067959189	0.006140918
PDRG1	0.093318702	3.2159E-26	0.054906097	0.019392372
MAFG	0.09415219	6.96685E-18	0.061476796	0.002262443
MAP3K11	0.094247963	1.18535E-12	0.08517983	0.000323206
UNC13B	0.09452515	1.19158E-23	0.075047547	0.048480931
GTF2H2	0.094662128	1.11496E-11	0.034586278	0.013574661
APOOL	0.094743555	5.04305E-14	0.060128511	0.026826115
RORC	0.094898103	1.07398E-08	0.089802454	0.026826115
NCOA6	0.09496621	2.40664E-37	0.051706902	0.036522301
FAM122B	0.095183118	3.34652E-10	0.045503027	0.026826115
CDCA8	0.095685068	8.40403E-30	0.071941023	0.002262443
CALR	0.095831317	1.56201E-12	0.058585078	0.017636487
MOGAT3	0.096132013	6.6752E-08	0.178969675	0.026826115
RBMY1A1	0.096267602	0.01881465	0.062199599	0.003878474
LGALS8	0.096462909	4.9926E-09	0.03512921	0.036522301
ERCC2	0.096545475	1.01793E-25	0.053962146	0.019392372
ABCC6	0.096736062	2.40204E-07	0.062922711	0.019392372
MBTD1	0.096786326	1.75488E-11	0.047669647	0.013574661
CEBPG	0.096799549	9.39654E-23	0.051786605	0.048480931
MTMR15	0.096900782	1.46626E-18	0.106724251	0.002262443
KIFAP3	0.097141106	5.1075E-21	0.035436927	0.036522301

PDZK1	0.097744733	5.04912E-28	0.029372413	0.036522301
ZFAND3	0.098106468	7.33126E-28	0.055544298	0.006140918
ATRN	0.098232934	4.9926E-09	0.056217049	0.006140918
DHX37	0.098264126	5.96634E-16	0.024627615	0.048480931
UBR5	0.098421645	3.60113E-19	0.058525116	0.019392372
PRPSAP1	0.099274814	7.95458E-20	0.070812444	0.013574661
DRAP1	0.099339038	4.8141E-21	0.046601146	0.006140918
ZNF142	0.099730526	5.92461E-17	0.070190814	0.006140918
PUS7	0.099956239	7.07803E-11	0.076843299	0.036522301
NIT1	0.100093803	8.47406E-13	0.077989892	0.000323206
TNPO3	0.100257751	1.34895E-30	0.093045304	0.039711134
SLC22A18	0.100271752	8.64023E-13	0.049052833	0.048480931
DOM3Z	0.100603579	6.36162E-11	0.062346382	0.000646412
RHEB	0.100953908	1.60158E-27	0.065608308	0.003725294
NUB1	0.101051468	6.68545E-33	0.044159584	0.048480931
TIGD6	0.101325864	2.44805E-32	0.040865065	0.002262443
RAD18	0.101454355	5.20497E-22	0.024278033	0.013574661
KEAP1	0.101463319	3.89828E-32	0.040428561	0.001292825
COPS5	0.101606391	1.61449E-15	0.022666034	0.036522301
TXLNA	0.10204224	1.03999E-15	0.051618017	0.013574661
POMGNT1	0.10205611	3.62741E-25	0.073452157	0.036522301
ERAL1	0.102373624	7.54197E-20	0.026206411	0.036522301
HSPA4	0.102624586	6.41985E-35	0.066122016	0.003878474
WDR12	0.102756283	1.24201E-34	0.043207843	0.036522301
SQSTM1	0.103052987	2.5988E-36	0.070069704	0.00937298
MED24	0.103267133	9.7451E-38	0.05762409	0.001292825
KIF1B	0.103310906	2.57528E-16	0.083344148	0.000323206
DEDD	0.103380545	2.79312E-20	0.026854826	0.036522301
RNF31	0.103466079	9.04048E-13	0.049918466	0.00937298
SLC17A4	0.103599901	6.10524E-08	0.105163457	0.013188555
NUPL2	0.103704044	1.27687E-20	0.044212909	0.036522301
SOX12	0.104189058	2.37726E-15	0.027179669	0.048480931
OPLAH	0.104237998	5.36719E-16	0.103934845	0.002262443
NUDT12	0.105128268	1.10457E-13	0.046363987	0.036522301
UBE2O	0.105540498	7.8119E-22	0.035590658	0.019392372
ABHD4	0.105756939	1.70831E-37	0.067821498	0.019392372
RNF14	0.105921692	5.65062E-15	0.034345075	0.013574661
SLC4A2	0.106106019	2.81926E-18	0.110535356	0.002262443
LYSMD4	0.106785211	1.28042E-23	0.051179935	0.036522301
NPAS2	0.107015374	4.38843E-12	0.094919556	0.036522301
REPIN1	0.107237764	3.05792E-37	0.049306583	0.001292825
EIF2AK1	0.107888763	3.96867E-40	0.027369788	0.009763047
CHD1L	0.10834033	4.2764E-21	0.057760286	0.048480931
PSMB4	0.109632951	3.84181E-46	0.024418327	0.026826115
CDC23	0.110200591	7.57051E-39	0.060396025	0.002262443
XPR1	0.110889432	8.79636E-15	0.069105976	0.036522301
PLOD3	0.111223087	3.59994E-36	0.100136631	0.019392372
ANKRD54	0.111562244	3.46408E-20	0.056831658	0.001292825
TRIM31	0.111593056	6.07363E-06	0.046928132	0.013574661
POLR2K	0.112111383	1.2605E-29	0.046400405	0.013574661
DUSP3	0.112471582	7.99553E-14	0.078415996	0.002262443
MOV10	0.112472341	2.4243E-14	0.066227581	0.019392372
CABC1	0.11252032	2.10421E-09	0.064668681	0.013574661
ERMP1	0.112770834	6.38653E-34	0.072449474	0.048480931

MAP3K13	0.113421658	4.36098E-12	0.149002241	0.019392372
COIL	0.113529977	3.15802E-33	0.033433591	0.048480931
BAT3	0.113651638	1.81671E-23	0.046409528	0.036522301
ZNF34	0.113841983	1.10783E-17	0.038818605	0.048480931
XYLB	0.114136949	2.85917E-12	0.145314479	0.019392372
RGS14	0.114819138	9.7665E-21	0.061900129	0.026826115
MAP4K4	0.114912523	8.81329E-22	0.028710006	0.013574661
GOLT1B	0.114944091	2.50655E-23	0.055997191	0.036522301
APTX	0.114964413	4.09066E-23	0.037751278	0.026826115
CCDC15	0.115186657	1.22456E-20	0.064079945	0.026826115
NFE2L1	0.115212359	2.31335E-17	0.077081685	0.003878474
TRAF7	0.115213183	3.74247E-29	0.042203866	0.00937298
RFWD2	0.115298432	7.1729E-29	0.022161093	0.00937298
SETDB1	0.115315347	6.20521E-23	0.073858104	0.048480931
TIPRL	0.115417238	4.23861E-23	0.04124721	0.048480931
KIAA1522	0.115462671	3.0094E-23	0.077516917	0.013574661
KIAA0907	0.115727829	5.89799E-19	0.076320672	0.013574661
MTSS1	0.115749925	9.52134E-09	0.104797742	0.000646412
NRBP1	0.116076458	3.01743E-13	0.047430416	0.036522301
NEK2	0.116082449	7.25429E-37	0.081884226	0.002262443
SPIRE2	0.116162776	4.65063E-22	0.11587493	0.003878474
IPO9	0.116626478	2.0517E-36	0.052180019	0.006140918
TIMM44	0.11699393	1.04157E-17	0.094128772	0.002262443
ATP6V1C1	0.117267726	1.72461E-20	0.055480191	0.013574661
TMEM65	0.118047898	9.24592E-10	0.048470224	0.000323206
PRRC1	0.118150104	1.688E-40	0.040930071	0.001292825
WDR67	0.118322076	1.32827E-30	0.084405618	0.000323206
GPATCH2	0.119350873	1.8148E-18	0.057051587	0.003878474
YEATS2	0.119496563	4.73644E-15	0.07178994	0.002262443
EIF4EBP2	0.119976586	4.5707E-22	0.040621432	0.013574661
PIGT	0.12027646	3.16019E-27	0.032976477	0.013574661
SNTA1	0.120584867	4.54606E-19	0.052922082	0.026826115
VPS52	0.121028846	1.73868E-28	0.032856182	0.019392372
LARP1	0.121331936	1.05431E-45	0.03249722	0.00937298
BRD8	0.12136129	1.26719E-19	0.047764	0.002262443
DAP3	0.121765927	4.04222E-33	0.052903586	0.048480931
RAD21	0.12189499	1.606E-22	0.049552991	0.048480931
TCTA	0.122381704	4.70162E-20	0.072234646	0.002262443
TOP1MT	0.122752873	7.13238E-25	0.063349599	0.001292825
SAE1	0.123006788	2.2482E-24	0.043671347	0.036522301
ASPH	0.123111756	2.01064E-18	0.039695087	0.00937298
AP1M1	0.12513919	5.5174E-26	0.029007544	0.036522301
LIN37	0.126065678	1.51325E-28	0.062293062	0.003878474
LRP11	0.126104733	2.68736E-28	0.063037554	0.006140918
MARVELD2	0.126481811	1.35843E-15	0.056805735	0.003878474
ZSCAN2	0.126937172	5.08254E-46	0.023389189	0.019392372
PHF14	0.127953271	2.97233E-30	0.037323262	0.026826115
LARS	0.128205653	3.14466E-45	0.052521417	0.026826115
MRPL55	0.128206441	2.25497E-38	0.035308447	0.048480931
LRRC14	0.128642172	2.42235E-22	0.056444222	0.006140918
RNF157	0.128999507	5.20471E-07	0.055324211	0.009763047
HLTF	0.129057238	1.79531E-29	0.056250573	0.003878474
ARMC1	0.129463051	1.18573E-11	0.040639746	0.019392372
SLC30A10	0.131516506	8.80406E-16	0.059558834	0.048480931

AGPAT5	0.132320158	1.69618E-08	0.12989768	0.000323206
EMX1	0.133234783	9.30152E-30	0.058280698	0.00937298
ISG20L2	0.133880539	2.5473E-31	0.041523157	0.019392372
AKT1S1	0.134687716	1.98043E-32	0.070959706	0.000323206
APH1A	0.13533226	7.12806E-33	0.042500024	0.019392372
COG7	0.135488516	5.5781E-22	0.031537354	0.036522301
TM7SF2	0.136081613	9.71682E-18	0.077250734	0.036522301
CTSA	0.136334319	1.08977E-24	0.067066407	0.013574661
KLF13	0.138034986	8.98029E-21	0.094355254	0.019392372
CA14	0.138271392	0.009952266	0.076700362	0.036522301
RECQL4	0.138625153	1.00153E-20	0.059046049	0.00937298
CDK2AP2	0.13902433	5.52628E-16	0.066401891	0.013574661
FBXL18	0.140227793	1.83851E-24	0.043562602	0.026826115
CDC42BPA	0.140893605	1.81548E-22	0.047706137	0.002262443
CDK5RAP2	0.140979738	4.85443E-28	0.078687585	0.00937298
ALKBH6	0.142326294	1.43109E-26	0.052767359	0.013574661
AKR1C3	0.142452073	7.09448E-43	0.088493022	0.006140918
SLC17A5	0.142639199	8.11908E-21	0.052455799	0.002262443
UBQLN4	0.143106775	2.85834E-38	0.069510237	0.001292825
H1F0	0.145950297	4.57348E-29	0.058796531	0.006140918
ZSCAN21	0.146496279	3.05786E-33	0.092775572	0.000646412
MRPL24	0.146758724	1.16674E-40	0.041903761	0.00937298
ZNF692	0.146955068	5.89573E-21	0.063817075	0.000646412
FAM54A	0.146996645	3.5277E-25	0.028681157	0.036522301
ATG10	0.147603137	4.10676E-28	0.058822241	0.001292825
PTCD1	0.148134691	2.50625E-45	0.092525939	0.013574661
NEDD4	0.149178088	7.68915E-13	0.035710932	0.026826115
CYB5R1	0.150512768	4.5707E-22	0.068117108	0.006140918
EPHX1	0.151214466	6.83914E-23	0.128040769	0.000323206
STIP1	0.151452345	6.747E-21	0.059204044	0.00937298
GRINA	0.152644304	1.73774E-22	0.097030679	0.000323206
KISS1	0.153059973	6.48E-20	0.045268297	0.003878474
CLPTM1	0.154180029	1.19168E-22	0.087649024	0.00186152
RPAP1	0.154469044	2.07494E-36	0.067666943	0.000646412
BPNT1	0.155303958	1.55866E-29	0.060794162	0.006140918
PPP2R5A	0.155499583	1.16094E-44	0.06650013	0.013574661
NSUN5	0.155515576	2.71904E-42	0.058883704	0.048480931
MCM8	0.155576494	2.1226E-37	0.037142224	0.013574661
FICD	0.156053068	3.01213E-22	0.077855243	0.006140918
RMND5B	0.157067596	1.57452E-28	0.071907601	0.001292825
ZNF219	0.157701356	2.81164E-36	0.061610759	0.006140918
CCT3	0.158687761	2.42121E-54	0.060975373	0.00937298
43892	0.159063856	1.07233E-22	0.041822858	0.048480931
AIFM2	0.161880629	3.03091E-29	0.098231444	0.001292825
INTS8	0.163006892	1.95643E-41	0.048540722	0.048480931
MORC2	0.163631138	3.80247E-39	0.086070679	0.001292825
EZH2	0.163709693	2.84076E-41	0.038410316	0.00937298
SMG7	0.163983077	3.34441E-21	0.087125261	0.000646412
NAGPA	0.1648437	9.62738E-40	0.045751379	0.013574661
PPME1	0.165031246	7.1186E-26	0.060549802	0.002262443
FBXO27	0.165387079	1.28229E-08	0.037819242	0.026826115
SLC25A39	0.167904029	2.19597E-44	0.054835671	0.006140918
CASC5	0.169494219	3.08133E-38	0.041898669	0.003878474
PSMD4	0.170376538	4.02548E-51	0.050313942	0.000646412

RNASEN	0.170721439	2.57397E-31	0.025822613	0.036522301
HFE2	0.172782532	3.61141E-12	0.085633379	0.00937298
SNX27	0.174885775	4.3453E-56	0.040317569	0.026826115
LRRC20	0.17544247	1.43787E-21	0.112512982	0.002262443
PREB	0.177250674	6.02958E-22	0.102139164	0.001292825
ZNF30	0.177663985	6.07096E-30	0.062349156	0.001292825
GPS1	0.177805388	2.6803E-27	0.050876856	0.048480931
GNPAT	0.179727005	2.87914E-45	0.056076043	0.036522301
SGOL2	0.18012335	2.39608E-34	0.04536529	0.001292825
ANKRD27	0.181909043	6.59405E-38	0.067411551	0.001292825
KHDRBS3	0.182509156	3.95523E-24	0.069998595	0.036522301
INTS7	0.182895408	1.83836E-35	0.085876642	0.013574661
ALKBH2	0.182963032	7.84697E-29	0.070899603	0.026826115
RMI1	0.184591177	1.12767E-29	0.037021846	0.013574661
KLHL12	0.186444029	2.58415E-51	0.066823575	0.000646412
TRIM24	0.18756276	6.75761E-40	0.050915025	0.013574661
PRC1	0.190206719	3.59682E-47	0.107385265	0.048480931
SCRIB	0.190300378	9.2273E-39	0.048253175	0.000323206
TXNRD1	0.191250292	3.29236E-30	0.093928525	0.048480931
THEM4	0.191788518	5.78172E-37	0.106676044	0.000323206
CD109	0.19377468	5.77447E-22	0.041418806	0.048480931
TUBG1	0.19718391	2.68483E-50	0.040781681	0.00937298
SMG5	0.199307051	4.32861E-31	0.091937972	0.003878474
CLN3	0.201055346	6.9843E-38	0.075728179	0.000646412
SERF1A	0.204105483	1.85199E-38	0.035631271	0.006140918
RBM24	0.209183525	1.70454E-28	0.046913812	0.003878474
ASPSCR1	0.213358571	1.50077E-21	0.066627405	0.013574661
TMEM106C	0.216037187	5.71474E-47	0.083268704	0.013574661
MFSD3	0.2188231	5.98387E-25	0.067807425	0.004402322
MANEAL	0.219015026	6.03086E-31	0.054960346	0.019392372
CDK5	0.22427595	7.39751E-38	0.093669058	0.013574661
SHARPIN	0.227464976	1.22375E-40	0.077594305	0.000323206
TMEM38B	0.228123343	1.66803E-40	0.048665613	0.000323206
SCAMP3	0.228658375	7.06548E-41	0.041010693	0.001292825
MAGEA1	0.229296285	1.08803E-16	0.202407138	0.048480931
PRCC	0.233739726	7.48966E-49	0.035494612	0.019392372
SRXN1	0.239633272	1.41412E-36	0.127265966	0.048480931
STEAP2	0.244678107	2.14537E-11	0.076801328	0.036522301
PEX6	0.247211558	1.02754E-25	0.108891779	0.00937298
GBA	0.255532013	2.19882E-50	0.065512505	0.001292825
ACSM1	0.256483618	1.00212E-16	0.144874578	0.013574661
PPP1R16A	0.257014301	1.17895E-41	0.107473294	0.00186152
TBCE	0.268452645	5.39861E-40	0.072932243	0.000323206
ATAD2	0.272398427	1.14358E-42	0.10494111	0.019392372
GPAA1	0.295401227	1.15085E-38	0.100867877	0.036522301
OSGIN1	0.29947907	3.37289E-22	0.059108026	0.036522301
C1QTNF3	0.315559632	1.1505E-22	0.020018371	0.048480931
HOXA10	0.317863232	8.01149E-16	0.020100091	0.036522301
SLC38A6	0.322085281	7.82707E-46	0.109003652	0.003878474
TP53I3	0.325964573	5.23832E-41	0.069884532	0.048480931
KIAA0101	0.330222685	4.94504E-38	0.026784535	0.036522301
NDC80	0.337001818	1.56742E-44	0.03995158	0.006140918
TPX2	0.345982457	5.11431E-41	0.045025144	0.026826115
AURKA	0.363506377	1.80501E-45	0.140450584	0.019392372

HOXA13	0.385180434	2.54796E-37	0.190914017	0.002262443
HMMR	0.391431624	2.38792E-54	0.151737171	0.003878474
BUB1	0.417162794	4.08986E-48	0.076069145	0.048480931
CENPF	0.492496803	1.9458E-56	0.111583823	0.003878474
CAP2	0.533946356	6.20219E-61	0.162348122	0.000646412
ASPM	0.542926941	1.55873E-55	0.197737659	0.013574661
AKR1B10	0.71763633	8.08533E-29	0.255305472	0.026826115
VTN	0.03	0.023563497	0.042535247	0.036522301
