# Supplementary material for: Identification of New Biomarker for Prediction of Hepatocellular Carcinoma Development in Early-Stage Cirrhosis Patients
Source: J Oncol. 2021 Jul 20;2021:9949492. doi: 10.1155/2021/9949492 (PMC8318773; doi:10.1155/2021/9949492)
Supplement: Supplementary Materials — Supplementary Figure A. The underlying pathological pathways by which the signature used to influence the development of HCC for early-stage liver cirrhosis patients. Supplementary Figure B. Schematic depicting the pathological pathways mediated by signature and inhibited by suggested bioactive compounds (HDAC inhibitors). Supplementary Material 1. DEGs between early HCC patients and cirrhosis patients identified by overlapping GSE63898 and GSE89377 datasets. Supplementary Material 2. DEGs identified by overlapping GSE63898, GSE89377, and GSE15654 datasets. Supplementary Material 3. HCC development related genes identified by univariate analysis in the patients of the training cohort. [file 9949492.f1.zip › 9949492.f1/Supplementary Material 2.pdf]

**DEGs identifed by overlapping GSE63898, GSE89377 and GSE15654 datatset**

| Gene | logFC of GSE63898 | adj.pValue | logFC of GSE89377 | adj.pValue | logFC of GSE15654 | adj.pValue |
|---|---|---|---|---|---|---|
| IQGAP3 | 0.063943022 | 0.000130443 | 0.234105194 | 0.030570207 | 0.133717788 | 0.029462358 |
| TMEM64 | 0.115106817 | 0.00000612 | 0.293691059 | 0.000845302 | 0.109977746 | 0.014974912 |
| CYP39A1 | -2.313555211 | 9.11E-45 | -0.849833756 | 0.012217446 | -0.156009471 | 0.01608967 |
| MAFG | 0.33006352 | 3.42E-13 | 0.280569518 | 0.000688132 | 0.294013663 | 0.00168757 |
| RBMY1A1 | 0.294998285 | 3.45E-06 | 0.256482375 | 0.001877255 | 0.144815527 | 0.043633198 |
| SEMA4D | -0.046049744 | 0.001190712 | -1.008620505 | 0.00023304 | -0.106758225 | 0.036299719 |
| DPP3 | 0.255058328 | 0.0000142 | 0.306513006 | 0.016198752 | 0.182729724 | 0.011787717 |
| AGPAT1 | 0.156296557 | 1.67E-11 | 0.190663397 | 0.002483969 | 0.186401402 | 0.02778079 |
| PPFIA1 | 0.246276395 | 0.00000432 | 0.128611337 | 0.143097317 | 0.160572246 | 0.038469423 |
| PI15 | 0.054472851 | 0.010330027 | 0.233683217 | 0.000989332 | 0.091242831 | 0.049096698 |
| SELP | -0.649639091 | 1.44E-28 | -0.224281951 | 0.014347518 | -0.188104313 | 0.006477635 |
| RNF31 | 0.37640877 | 4.64E-10 | 0.259512389 | 0.001096747 | 0.253051399 | 0.032080942 |
| AUP1 | 0.085229141 | 0.012937592 | 0.182216008 | 0.005179032 | 0.104150635 | 0.034034375 |
| BASP1 | -1.431183746 | 1.12E-27 | -0.854758531 | 6.21756E-05 | -0.246601542 | 0.014779706 |
| GNPAT | 1.006111753 | 1.08E-47 | 0.325663687 | 0.014016296 | 0.101354391 | 0.033635751 |
| HSD17B12 | -0.263799861 | 0.0000618 | -0.378412625 | 0.017881199 | -0.154060644 | 0.046688647 |
| CD1A | -0.018076161 | 0.730187008 | -0.186378136 | 0.041940705 | -0.22372636 | 0.035878529 |
| MGAT4B | 0.307806676 | 3.74E-13 | 0.361646178 | 0.001017964 | 0.158456135 | 0.025509463 |
| MRPL55 | 0.730985186 | 2.36E-35 | 0.215781259 | 0.045599067 | 0.216774457 | 0.002697261 |
| RPS3A | -0.259954594 | 1.47E-10 | -0.484445408 | 0.000110299 | -0.181672469 | 0.041930124 |
| ACSM1 | 1.015968501 | 2.07E-15 | 0.666077718 | 0.00560238 | 0.095945649 | 0.038916342 |
| YEATS2 | 0.518025757 | 3.42E-13 | 0.370993268 | 0.00633203 | 0.149758603 | 0.025982169 |
| HYOU1 | 0.30818407 | 8.28E-21 | 0.705658264 | 0.000955268 | 0.1630542 | 0.032463754 |
| COPS4 | -0.348496943 | 0.000000852 | -0.384723238 | 7.84916E-05 | -0.354788721 | 0.000614418 |
| TMSB4X | -0.435975954 | 4.75E-10 | -0.233436064 | 0.006373453 | -0.232132568 | 0.010081241 |
| PARP10 | 0.221303172 | 5.88E-08 | 0.353214769 | 0.000605653 | 0.118181394 | 0.01784123 |
| RECK | -0.305483 | 0.000000004 | -0.440572022 | 0.000152286 | -0.190415097 | 0.028633211 |
| NSUN3 | 0.132568964 | 0.006242851 | 0.224080403 | 0.000451027 | 0.070068364 | 0.036939403 |
| RBM28 | 0.161850327 | 0.00014115 | 0.445234695 | 0.00047058 | 0.21725827 | 0.003053423 |
| UTP6 | 0.222283921 | 0.000115372 | 0.291426817 | 0.007389894 | 0.139314525 | 0.032463754 |
| MXD3 | 0.130280065 | 0.000013 | 0.337703048 | 1.22651E-05 | 0.072576967 | 0.048552755 |
| ZSCAN1 | 0.100000749 | 0.0000041 | 0.193231668 | 0.016978728 | 0.103716771 | 0.00509603 |
| AP3D1 | 0.384128805 | 3.79E-10 | 0.190351634 | 0.004530065 | 0.205494102 | 0.008245069 |
| CCND3 | -0.147621251 | 0.0000502 | -0.800124054 | 0.000256042 | -0.168808644 | 0.046167241 |
| OGFR | 0.099596754 | 0.004615097 | 0.347271347 | 0.001911748 | 0.452025517 | 0.000113598 |
| NPLOC4 | 0.293116136 | 6.43E-20 | 0.822261569 | 5.8608E-05 | 0.446410549 | 0.012522296 |
| KIAA0556 | 0.110882868 | 0.00000159 | 0.306814081 | 0.006742475 | 0.152534026 | 0.013564658 |
| UPF1 | 0.236921254 | 0.00000296 | 0.459360213 | 0.003682304 | 0.158343521 | 0.03443703 |
| GSTM3 | -0.110569873 | 0.00751178 | -1.112720963 | 0.000245221 | -0.370178325 | 0.000405114 |
| EEF1G | -0.444242506 | 0.0000836 | -0.709141874 | 0.000995296 | -0.308164852 | 0.018898147 |
| EMX1 | 0.655662924 | 2.90E-24 | 0.238270754 | 0.000933271 | 0.077710029 | 0.037371171 |
| LILRB5 | -0.428948441 | 2.2E-48 | -0.469029712 | 0.001511326 | -0.148465505 | 0.021715144 |
