# Supplementary material for: Identification of New Biomarker for Prediction of Hepatocellular Carcinoma Development in Early-Stage Cirrhosis Patients
Source: J Oncol. 2021 Jul 20;2021:9949492. doi: 10.1155/2021/9949492 (PMC8318773; doi:10.1155/2021/9949492)
Supplement: Supplementary Materials — Supplementary Figure A. The underlying pathological pathways by which the signature used to influence the development of HCC for early-stage liver cirrhosis patients. Supplementary Figure B. Schematic depicting the pathological pathways mediated by signature and inhibited by suggested bioactive compounds (HDAC inhibitors). Supplementary Material 1. DEGs between early HCC patients and cirrhosis patients identified by overlapping GSE63898 and GSE89377 datasets. Supplementary Material 2. DEGs identified by overlapping GSE63898, GSE89377, and GSE15654 datasets. Supplementary Material 3. HCC development related genes identified by univariate analysis in the patients of the training cohort. [file 9949492.f1.zip › 9949492.f1/Supplementary Material 3.pdf]

HCC development related genes identified by univariate analysis in the patients of the training cohort

id	HR	HR.95L	HR.95H	pvalue
SEMA4D	0.149725684	0.043747221	0.512438961	0.002486433
RBM28	4.734492812	1.7205986	13.02768826	0.002605948
PARP10	4.227660069	1.585852283	11.27034961	0.003955416
OGFR	2.098414132	1.197387841	3.677456641	0.009617614
RPS3A	0.385080233	0.186559563	0.794849554	0.009853547
AGPAT1	2.844571174	1.256408444	6.440250542	0.012160793
TMSB4X	0.435440664	0.223312688	0.849072097	0.014680512
COPS4	0.475612851	0.260608178	0.867998794	0.015470141
DPP3	1.983011793	1.135865454	3.461973209	0.01603751
NPLOC4	1.498549114	1.074055929	2.090812392	0.017294497
MGAT4B	2.583257654	1.170896771	5.699238626	0.018735509
LILRB5	0.36709218	0.158525439	0.850063366	0.019328692
YEATS2	1.863027509	1.035571896	3.351647058	0.037836313
HYOU1	1.811716852	1.016154461	3.230136832	0.043979976
UTP6	2.220455258	0.987876012	4.990931547	0.053551735
RECK	0.552207826	0.293927638	1.037444063	0.064934524
GSTM3	0.675190022	0.436464088	1.044488148	0.077660428
AUP1	2.764583334	0.888085868	8.606060838	0.079239667
MAFG	1.515661201	0.934237029	2.458935797	0.09209948
RBMY1A1	1.633103874	0.895564727	2.978040763	0.109570981
MRPL55	1.669113017	0.87818194	3.172393026	0.11793339
RNF31	1.438959304	0.903171504	2.292592125	0.125670045
IQGAP3	1.676870169	0.863611848	3.255969184	0.126796685
BASP1	0.653999435	0.374704351	1.141473962	0.135089106
ACSM1	2.412180457	0.741731144	7.844641013	0.14335279
CCND3	0.619355472	0.317446094	1.208397921	0.160061428
PPFIA1	1.689488498	0.78479044	3.637112839	0.180079422
CD1A	0.710680941	0.420080254	1.20231169	0.202967648
CYP39A1	0.576693956	0.246817288	1.34745796	0.203645497
EEF1G	0.802033665	0.570363011	1.127804552	0.204646602
HSD17B12	0.766757515	0.491545994	1.196057123	0.241696315
AP3D1	1.444749952	0.778036482	2.682782195	0.243952154
UPF1	1.426918924	0.7774636	2.618897677	0.251176219
NSUN3	0.540234743	0.172937598	1.687623636	0.289370914
ZSCAN1	1.400081961	0.66264385	2.958194657	0.37791452
GNPAT	1.315075193	0.536523177	3.223388732	0.54932538
PI15	1.131371428	0.477915488	2.678300537	0.77891766
MXD3	1.04853933	0.400714799	2.743683864	0.923061672
EMX1	0.950514911	0.304783094	2.964333037	0.930310306
SELP	0.978993277	0.554213309	1.729348288	0.941700052
TMEM64	0.977788958	0.391714361	2.440735753	0.961616006
